# Rheology-Guided PHB/PBAT/MgO Nanocomposites for FDM Printing with Balanced Mechanical and Degradation Performance

**DOI:** 10.1021/acsomega.6c08431

**Published:** 2026-09-11

**Authors:** Evy Aracely Ortiz, Montana Thomas Hance, Aboulfazl Barati

**Affiliations:** Center for Materials and Manufacturing Sciences, Department of Chemistry and Physics, 8025 Troy University, Troy, Alabama 36082, United States

## Abstract

Biodegradable poly­(3-hydroxybutyrate) (PHB)/poly­(butylene adipate-*co*-terephthalate) (PBAT) blends are promising for fused deposition modeling (FDM), but their application is limited by phase immiscibility and inadequate melt stability. This study investigates the effects of reactive compatibilization with Joncryl ADR and MgO nanoparticle incorporation on the rheological behavior, FDM processability, mechanical performance, thermal behavior, morphology, and degradation of PHB/PBAT blends. Reactive compatibilization increased melt elasticity, complex viscosity, and relaxation time, accompanied by improved filament stability and FDM printability. The PHB/PBAT/J0.3 formulation exhibited the best mechanical performance, reaching a tensile strength of 17.73 MPa, an elongation at break of 705%, and a toughness of 78.66 MPa, corresponding to increases of approximately 86, 45, and 116%, respectively, relative to the uncompatibilized PHB/PBAT (25:75) blend. Thermal and morphological analyses showed formulation-dependent changes in crystallization, thermal stability, and phase morphology, while MgO incorporation influenced both mechanical and degradation behavior. In vitro testing in phosphate-buffered saline at 37 °C showed progressive degradation over 42 days. Overall, the results demonstrate that controlling melt viscoelasticity through reactive modification provides a practical route to balancing FDM processability, mechanical performance, and degradation behavior in biodegradable PHB/PBAT-based materials.

## Graphical abstract

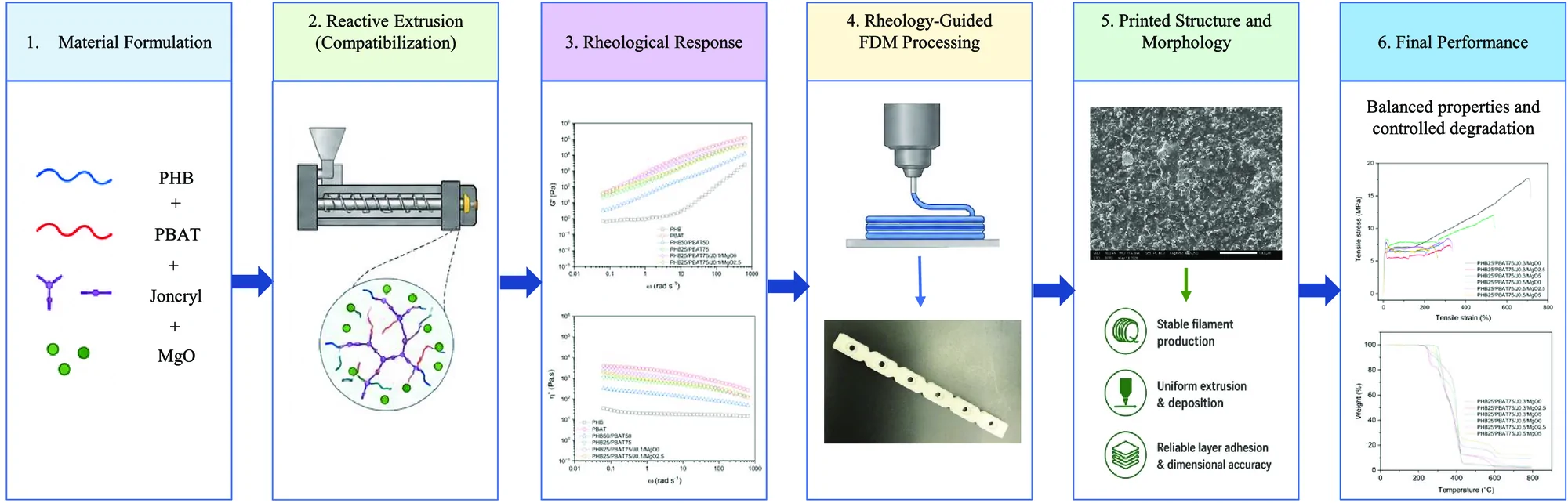

## Introduction

Orthopedic fixation systems, including plates, screws, and rods, are widely used to stabilize bone fractures and repair critical-sized defects. Although metallic implants provide excellent mechanical strength, their stiffness greatly exceeds that of native bone. This mismatch reduces physiological load transfer, promotes stress shielding, and can lead to bone resorption over time, ultimately compromising the long-term implant performance. Metallic implants also frequently require secondary removal surgery after healing, increasing both patient burden and healthcare costs.^1–4^ These limitations have driven increasing interest in biodegradable polymer-based implants that provide temporary mechanical support while gradually degrading in vivo, eliminating the need for implant removal and supporting natural bone regeneration.^5–8^

Poly­(3-hydroxybutyrate) (PHB) has attracted considerable attention for biodegradable orthopedic devices because of its biocompatibility, complete biodegradability into non-toxic products, and reported bioactivity, including piezoelectric behavior that may promote osteogenesis.^9,10^ Despite these advantages, its high crystallinity makes PHB inherently brittle, resulting in limited ductility and poor impact resistance.^11–13^ PHB also has a narrow processing window and readily undergoes thermal and hydrolytic degradation during melt-processing. These characteristics reduce melt strength, destabilize flow during extrusion, and complicate FDM printing.^14,15^ Material design strategies must therefore improve the processability without compromising mechanical performance.

Blending PHB with poly­(butylene adipate-*co*-terephthalate) (PBAT) provides an effective strategy for improving toughness and melt processability while maintaining biodegradability.^16–19^ Previous studies have consistently shown that increasing the PBAT content enhances ductility, impact resistance, and melt processability while reducing the brittle behavior of PHB.^20–27^ For example, Jeffri et al. reported a substantial improvement in ductility with increasing PBAT content, whereas Cai et al. showed that PBAT also modifies the crystallization behavior and thermal response of PHB/PBAT blends.^24,26^ However, PHB and PBAT are inherently immiscible, resulting in phase-separated morphologies, weak interfacial adhesion, and inefficient stress transfer between the two phases.^28,29^ This immiscibility leads to heterogeneous microstructures and unstable interfaces, limiting the mechanical performance and melt processability, particularly under the complex flow conditions encountered during melt extrusion and FDM printing.^30–33^

Reactive epoxy-functional chain extenders, such as Joncryl ADR, are widely used to improve the processing and mechanical performance of immiscible polyester blends. Previous studies suggest that these improvements arise from a combination of chain extension, branching reactions, and enhanced interfacial interactions rather than interfacial compatibilization alone.^34–36^ Andrzejewski et al. reported increases in storage modulus and complex viscosity after incorporating multifunctional epoxy chain extenders, indicating changes in melt viscoelasticity.^37^ Joncryl ADR 4468 was selected as the chain extender because its multifunctional epoxy groups can react with both hydroxyl and carboxyl end-groups of polyester chains during melt processing, enabling simultaneous modification of PHB and PBAT within a single reactive-blending step. Compared with chain extenders based on more selective reactive chemistries, such as diisocyanates or anhydride-functional compounds, multifunctional epoxy chain extenders offer broad reactivity toward polyester end-groups and can produce substantial changes in melt viscoelasticity at relatively low concentrations.^38–41^ This combination is particularly relevant to PHB/PBAT blends, where the objective is not only to increase molecular connectivity but also to modify the interfacial and melt response of the immiscible phases while limiting the additive content. Joncryl ADR 4468 was therefore selected because its multifunctional epoxy chemistry provides a practical route to increasing melt strength and elasticity under conventional reactive extrusion conditions, properties that are directly relevant to filament formation and FDM processing. Nofar and Park further associated these rheological changes with improved morphological stability during melt processing.^42^ Collectively, these findings suggest that reactive compatibilization modifies melt rheology and morphology, leading to improved processing stability and mechanical performance in biodegradable polyester blends.^41,43^ Despite these advances, the relationship among reactive compatibilization, melt rheology, and FDM processing remains only partially understood. Most studies have focused on mechanical performance or phase morphology, whereas relatively few have explored how changes in melt rheology relate to filament formation and printability under practical processing conditions.^44–47^

Magnesium oxide (MgO) has attracted considerable attention as a functional filler for biodegradable orthopedic materials because it can influence both biological and processing-related properties.^48–50^ Previous studies have shown that MgO can regulate degradation kinetics by neutralizing acidic degradation products while enhancing bioactivity, osseointegration, and antibacterial performance.^51^ Nagchowdhury et al. reported that MgO-containing biodegradable nanocomposites promoted apatite formation and improved osteogenic potential, whereas Wetteland et al. demonstrated that MgO nanoparticles moderated hydrolytic degradation through their buffering effect.^52,53^ Canales et al. further showed that MgO altered the crystallization behavior and mechanical response of biodegradable polyester systems by influencing chain mobility and polymer–filler interactions.^54^ Although MgO has been shown to improve stiffness and antibacterial activity, excessive filler loading can promote particle agglomeration and reduce ductility.^55^ Overall, these studies establish the role of MgO in controlling degradation, crystallization, bioactivity, and mechanical performance in biodegradable polymer systems.^56–60^ Its influence on melt rheology, however, remains much less understood, particularly with respect to viscoelastic behavior, flow stability, and extrusion performance during additive manufacturing.

Fused deposition modeling (FDM) has emerged as a versatile platform for fabricating patient-specific orthopedic devices with complex geometries and controlled porous architectures.^61–64^ Successful FDM processing of biodegradable polymers depends strongly on melt rheology, including viscosity, shear thinning behavior, and viscoelastic recovery, which influence filament extrusion stability, interlayer adhesion, dimensional accuracy, and defect formation.^65,66^ However, the development of biodegradable formulations for material extrusion printing has often emphasized composition and final solid-state properties, with less attention to how formulation-induced changes in melt viscoelasticity translate into practical processing behavior.^67–69^

Recent studies of PBAT-containing systems illustrate both the opportunities and limitations of this approach. Studies of PLA/PBAT blends have shown that incorporation of PBAT can improve ductility and support successful 3D and 4D printing while also demonstrating that blend composition strongly affects interfacial quality, deformation behavior, and the resulting printed structure.^70^ The incorporation of functional nanoparticles, such as Fe_3_O_4_, into PLA/PBAT systems has further extended these materials toward magnetically responsive shape-memory applications, but these studies also demonstrate the importance of controlling filler loading and dispersion to avoid agglomeration and deterioration of material performance.^71^ Recent work on direct pellet printing of PBAT has likewise demonstrated that this highly ductile biodegradable polyester can be processed by material extrusion without first producing conventional filament.^72^ More broadly, advances in magnetically responsive biodegradable 4D-printed systems have highlighted the importance of coordinating matrix selection, functional filler incorporation, processing conditions, and final functional performance rather than optimizing these factors independently.^73^ These studies demonstrate the versatility of PBAT-containing materials for additive manufacturing but have primarily focused on PLA/PBAT systems, shape-memory functionality, functional filler incorporation, or alternative feedstock strategies. They provide comparatively less insight into how reactive modification and nanoparticle incorporation alter the melt viscoelasticity of PHB/PBAT blends and how these rheological changes relate to filament formation and FDM processing.

This distinction is important because improvements in solid-state mechanical or functional properties do not necessarily correspond to a melt response that is suitable for stable filament extrusion and printing. Although reactive chain extension and nanoparticle incorporation have been widely investigated in biodegradable polyester systems, rheological characterization is often discussed separately from practical processing behavior.^74–76^ In PHB/PBAT systems, in particular, the combined effects of reactive chain extension and MgO incorporation on melt viscoelasticity, filament formation, and the resulting mechanical and degradation behavior remain insufficiently resolved. The present study addresses this gap by using melt rheology as a central framework to relate formulation-dependent changes in PHB/PBAT/MgO systems to filament stability, FDM processing, mechanical performance, thermal behavior, morphology, and degradation. By systematically varying blend composition, Joncryl content, and MgO loading, the study examines how changes in viscoelastic response translate into processing and material performance outcomes, with particular attention to the trade-offs among melt behavior, mechanical integrity, and degradation-related functionality.

## Materials and Methods

Poly­(3-hydroxybutyrate) (PHB) (Biocycle 1000, PHB Industrial SA, Brazil) and poly­(butylene adipate-*co*-terephthalate) (PBAT) (Ecoflex F Blend C1200, BASF, Germany) were used as the biodegradable polymer matrices. Joncryl ADR 4468 (molecular weight = 7250 g mol^–1^; epoxy equivalent weight = 310 g mol^–1^, BASF, Germany) served as a multifunctional epoxy-functional chain extender. Magnesium oxide (MgO) nanoparticles (20–50 nm, Sigma-Aldrich, USA) were used as the inorganic filler, and Irganox 1010 (BASF, Germany) was added to minimize thermooxidative degradation during melt processing.

All polymer pellets were vacuum-dried at 60–70 °C for 24 h before processing. Formulations are listed in Table 1. Reactive melt blending was performed using a co-rotating twin-screw extruder (Thermo Scientific Process 11; screw diameter, 11 mm; L/D = 40). The barrel temperature was maintained between 130 and 180 °C, and the screw speed was fixed at 50 rpm. PHB, PBAT, Joncryl, and MgO nanoparticles were dry blended before extrusion. The residence time was approximately 3 min to promote reactions between epoxy groups and polymer chain ends while minimizing thermal degradation. The extrudates were air-cooled, pelletized, and stored in sealed containers before filament fabrication.

**Table 1:** Formulation of PHB/PBAT/MgO Nanocomposite

sample	PHB (wt %)	PBAT (wt %)	Joncryl ADR (wt %)	MgO (wt %)	Irganox 1010 (wt %)
PHB	99.80	0	0	0	0.2
PHB/PBAT 75:25	74.25	24.75	0	0	0.2
PHB/PBAT 50:50	49.50	49.50	0	0	0.2
PHB/PBAT 25:75	24.75	74.25	0	0	0.2
PBAT	0	99.80	0	0	0.2
PHB/PBAT/J 25:75:0.1	74.18	24.72	0.1	0	0.2
PHB/PBAT/J/MgO 25:75:0.1:2.5	72.30	24.10	0.1	2.5	0.2
PHB/PBAT/J/MgO 25:75:0.1:5	70.43	23.48	0.1	5.0	0.2
PHB/PBAT/J 25:75:0.3	74.03	24.67	0.3	0	0.2
PHB/PBAT/J/MgO 25:75:0.3:2.5	72.15	24.05	0.3	2.5	0.2
PHB/PBAT/J/MgO 25:75:0.3:5	70.28	23.43	0.3	5.0	0.2
PHB/PBAT/J 25:75:0.5	73.88	24.62	0.5	0	0.2
PHB/PBAT/J/MgO 25:75:0.5:2.5	72.00	24.00	0.5	2.5	0.2
PHB/PBAT/J/MgO 25:75:0.5:5	70.13	23.37	0.5	5.0	0.2

Filaments were produced using a Filabot EX2 single-screw extruder after drying the compounded pellets under vacuum at 60-70 °C for 24 h. Extrusion conditions were optimized to maintain stable melt flow while minimizing thermal degradation of PHB. Barrel and nozzle temperatures were maintained between 165 and 180 °C, the screw speed ranged from 5 to 15 rpm, and the extruded filament was cooled under controlled airflow before spooling. Processing conditions were adjusted to obtain a target filament diameter of 1.75 ± 0.1 mm.

The filament diameter was controlled through synchronization of the extrusion rate, haul-off speed, and cooling rate. A digital caliper was used to ensure dimensional consistency. Filament quality was assessed from diameter uniformity, surface appearance, flexibility, spoolability, and the absence of visible defects, such as bubbles or surface irregularities.

Printing parameters, including nozzle temperature (210–230 °C), bed temperature (50–60 °C), printing speed (40–70 mm s^–1^), and layer height (0.1–0.3 mm), were selected to ensure stable printing of all formulations. All specimens were printed flat on the build platform, with the longitudinal axis of the specimen parallel to the build plane. A ±45° raster orientation with a rectilinear infill pattern was used for all formulations. These printing parameters were kept constant across all specimens to minimize processing-induced variability when comparing the different formulations. Standard tensile specimens (ASTM D638) were printed for mechanical characterization. Printability was assessed qualitatively by comparing extrusion continuity, filament deposition, dimensional consistency, layer adhesion, and the presence of visible printing defects under identical printing conditions for all formulations.

### Characterization Methods

Fourier transform infrared (FTIR) spectra were collected using a Spectrum Two spectrometer (PerkinElmer). Spectra were recorded with a resolution of 4 cm^–1^ over 32 scans.

Tensile properties were measured at room temperature using an Instron 5943 universal testing machine in accordance with ASTM D638. Specimens had a gauge length of 15 mm, a width of 4 mm, and a thickness of 0.5 mm. Tests were performed at a crosshead speed of 5 mm min^–1^ using a 10 kN load cell. Tensile strength, Young’s modulus, elongation at break, and toughness were determined from the resulting stress-strain data. Toughness was calculated by numerical integration of the area under the engineering stress-strain curve from the onset of loading to fracture. Because engineering strain is dimensionless, the integrated area retains the units of stress and is therefore reported in MPa. Equivalently, 1 MPa corresponds to 1 MJ m^–3^; thus, the reported toughness represents the energy absorbed per unit volume of material prior to fracture. Mechanical testing was performed using five independent specimens for each formulation (*n* = 5), and the results are reported as mean ± standard deviation. Statistical differences among formulations were evaluated using one-way analysis of variance (ANOVA), followed by Tukey’s honestly significant difference (HSD) post-hoc test for multiple comparisons. Differences were considered statistically significant at *p* < 0.05.

Unnotched tensile-impact tests were conducted using a Zwick/Roell HIT25P pendulum impact tester equipped with a 2.7 J pendulum according to ASTM D1822-S. Type S specimens had a total length of 63.5 mm, a grip separation of 25.4 mm, a narrow-section width of 3.18 ± 0.03 mm, and a thickness of 0.5 mm, consistent with the standard specimen geometry. Unnotched specimens were selected to evaluate the intrinsic energy-absorption capability of the materials without introducing an artificial stress concentrator. The selected pendulum energy was sufficient to ensure complete specimen fracture without reaching the instrument energy limit. Results are reported as the mean ± standard deviation of at least five independent specimens per formulation.

Thermogravimetric analysis (TGA) was performed using a TGA550 thermogravimetric analyzer (TA Instruments, USA) under a nitrogen atmosphere. Samples were heated from 30 to 800 °C at a heating rate of 10 °C min^–1^. Differential scanning calorimetry (DSC) was performed using a DSC 250 (TA Instruments, USA) under a nitrogen atmosphere (50 mL min^–1^). Samples (5–10 mg) were heated from room temperature to 280 °C at 10 °C min^–1^ and held for 2 min to erase thermal history. The samples were then cooled to room temperature at the same rate, followed by a second heating cycle to 280 °C. The glass transition temperature (*T*_g_), melting temperature (*T*_m_), and melting enthalpy (Δ*H*_m_) were determined from the second heating cycle, whereas the crystallization temperature (*T*_c_) and crystallization enthalpy (Δ*H*_c_) were obtained from the cooling cycle.

Rheological properties were characterized by small-amplitude oscillatory shear (SAOS) using a DHR HR-30 rheometer (TA Instruments, USA) equipped with 25 mm parallel plates. Measurements were performed at 175 °C. Strain sweep tests were first conducted to identify the linear viscoelastic (LVE) region of each formulation. A strain amplitude of 5% was subsequently used for all frequency sweep measurements, which were carried out over a frequency range of 0.01–100 Hz, corresponding to an angular frequency range of 0.063–628 rad s^–1^. Because only one frequency-sweep measurement was performed for each formulation, experimental standard deviations could not be determined for the rheological data. Accordingly, the rheological comparisons are interpreted on the basis of overall frequency-dependent trends rather than small differences between individual data points.

Fracture surfaces were examined by using field-emission scanning electron microscopy (FESEM, JSM-IT710HR/LV, JEOL, Japan). Samples were mounted on aluminum stubs by using conductive carbon tape and sputter-coated with gold (Smart Coater, JEOL, Japan) before imaging. Micrographs were acquired at an acceleration voltage of 10 kV.

In vitro degradation was evaluated in phosphate-buffered saline (PBS, pH 7.2 ± 0.2) at 37 ± 1 °C for 42 days. Rectangular specimens (approximately 5 × 5 × 0.5 mm) were vacuum-dried before testing, and their initial dry weight (W_0_) was recorded. At predetermined intervals (7, 14, 21, 28, 35, and 42 days), specimens were removed, rinsed with deionized water, and vacuum-dried at room temperature to a constant weight before the final dry weight (*W*_t_) was measured. Mass loss was calculated using eq ([Disp-formula eq1])

1 $$m\mathrm{ass}\;\mathrm{loss}\;\left(\%\right)=\left(W_{0}-W_{t}\right)/W_{0}\times 100$$

The pH of the degradation medium was monitored periodically by using a calibrated digital pH meter (FiveEasy F20, Mettler Toledo). The degradation protocol followed ASTM F1635 and relevant ISO 10993-based procedures for biodegradable polymers.

## Results and Discussion

### Chemical Analysis

FTIR spectroscopy was used to examine the chemical structure and reactive interactions in neat PHB, PBAT, Joncryl, PHB/PBAT blends, and PHB/PBAT/MgO nanocomposites.

The proposed reaction pathways for the reactive compatibilization of PHB/PBAT blends with Joncryl ADR during melt blending are illustrated in Figure 1. Epoxy groups in Joncryl ADR can react with terminal carboxyl and hydroxyl groups of polyester chains, leading to chain extension, long-chain branching, and enhanced interfacial interactions, as proposed in previous studies.

**Figure 1:**
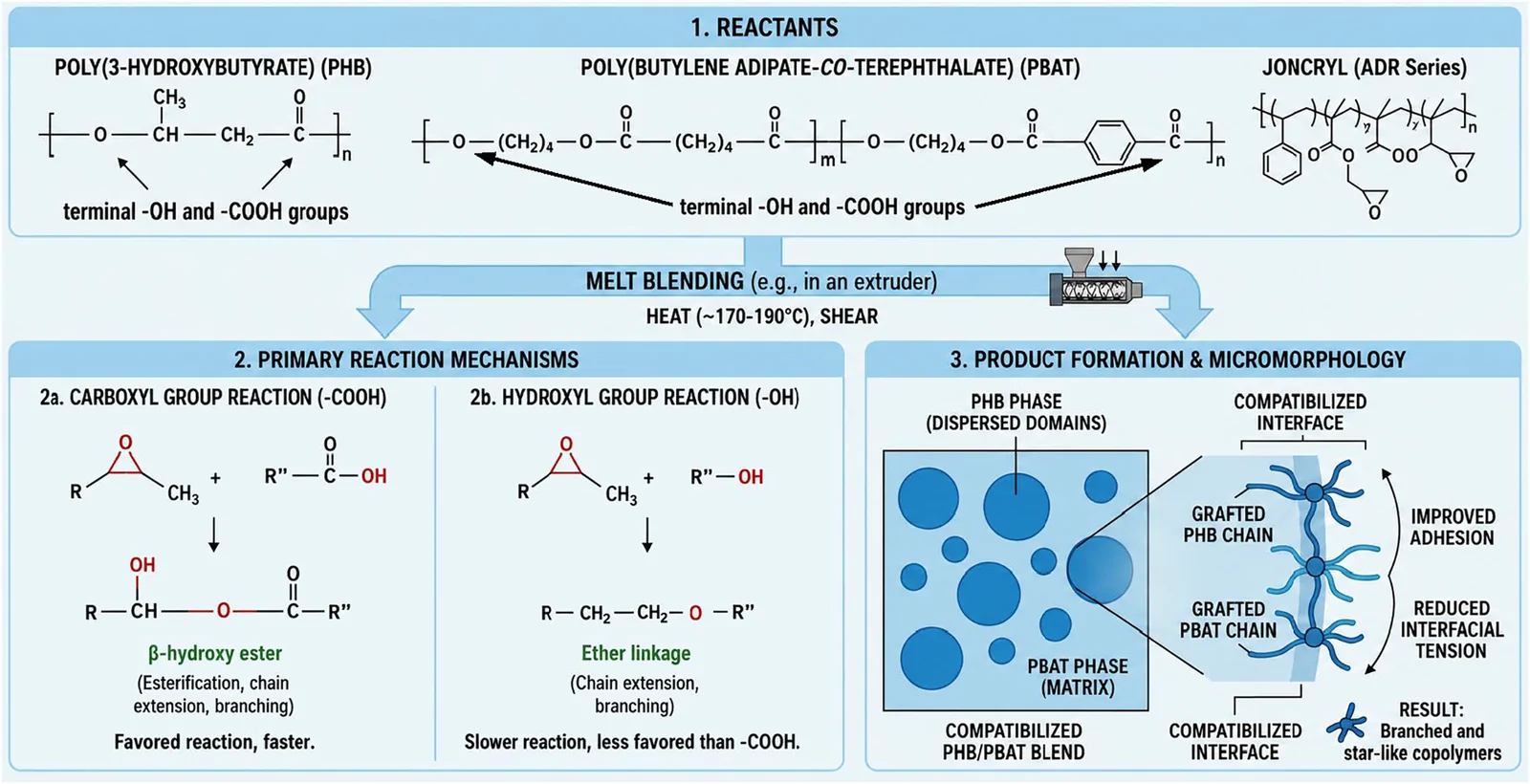
Schematic illustration of the principal reaction pathways during the reactive compatibilization of PHB/PBAT blends with the multifunctional epoxy chain extender Joncryl ADR.

The FTIR spectrum of PHB (Figures 2a and S1a) exhibited a strong ester carbonyl (C=O) absorption at ∼1720–1725 cm^–1^, together with characteristic C–H stretching bands at 2850–2975 cm^–1^ and C–O–C vibrations between 1050 and 1270 cm^–1^.^77^ The relatively sharp carbonyl band is consistent with the higher crystallinity of PHB. In contrast, PBAT exhibited a broader carbonyl band (∼1710–1720 cm^–1^) arising from aliphatic and aromatic ester groups, along with aromatic ring vibrations at ∼1500–1600 cm^–1^.^78^ This broader band is consistent with the lower crystallinity and greater chain mobility of PBAT.

**Figure 2:**
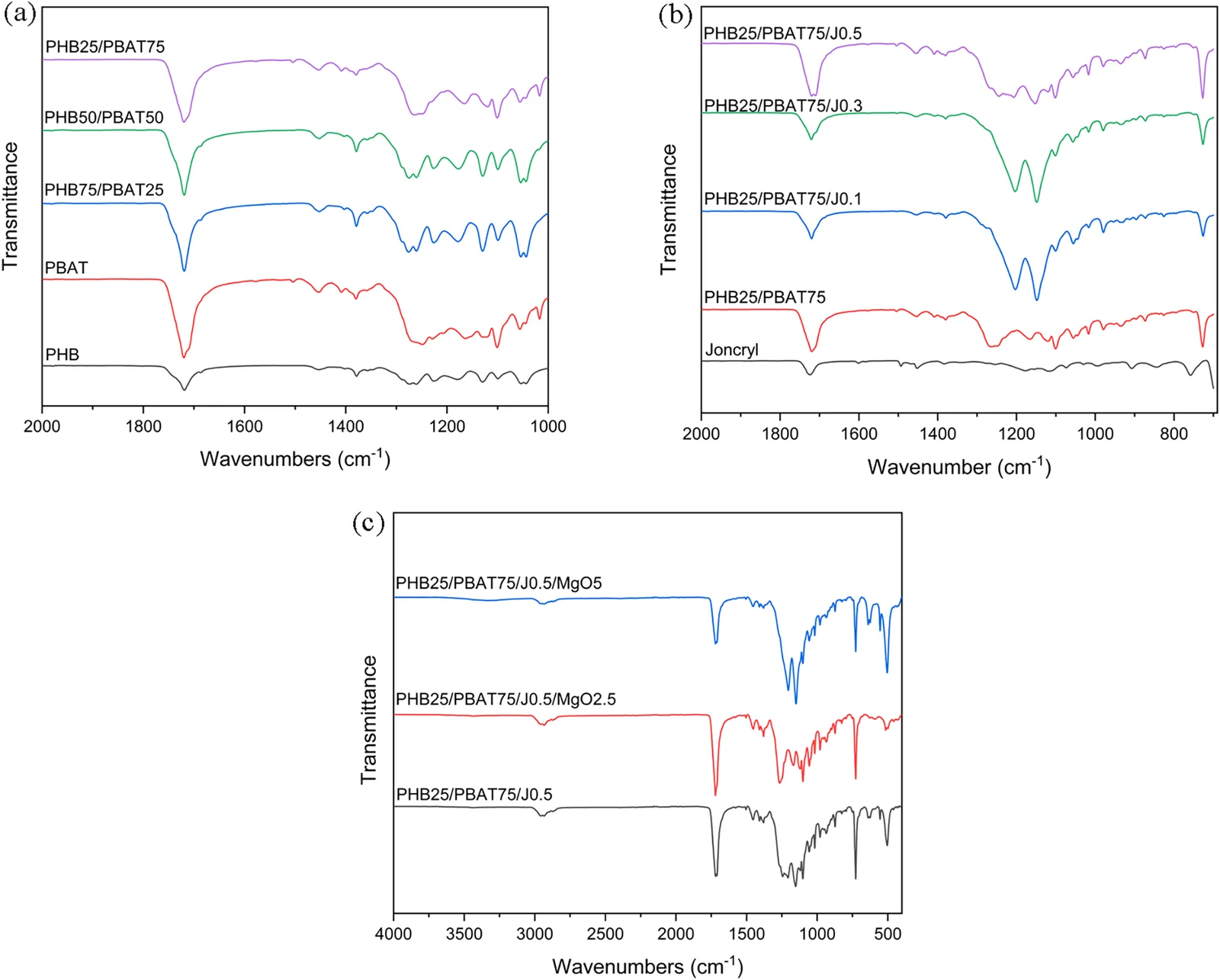
FTIR spectra of (a) neat PHB, PBAT, and PHB/PBAT blends with different blend ratios, (b) Joncryl and PHB/PBAT (25:75) blends containing different Joncryl contents, and (c) PHB/PBAT/J0.5 nanocomposites with varying MgO loadings.

The FTIR spectrum of Joncryl ADR displayed characteristic epoxy bands at ∼910 and ∼840–850 cm^–1^, corresponding to epoxy ring vibrations (Figures 2b and S1b). These bands are associated with epoxy groups that can undergo ring-opening reactions with terminal hydroxyl and carboxyl groups during reactive melt blending, as illustrated in Figure 1.^37,42,43^

The compatibilized PHB/PBAT/MgO nanocomposites exhibited noticeable spectral changes after reactive blending (Figure 2c). The reduced intensity of the epoxy band at ∼910 cm^–1^ indicates consumption of epoxy groups during processing, consistent with epoxy ring-opening reactions involving terminal hydroxyl and carboxyl groups. Slight broadening of the carbonyl region (∼1715–1725 cm^–1^) was also observed, suggesting changes in the local chemical environment associated with reactive compatibilization.

Changes in the C–O–C region (1100–1250 cm^–1^) are also consistent with reactive interactions occurring during melt blending. The observed spectral changes are consistent with modifications in the chemical environment of the polyester matrix expected during the epoxy-mediated reactive processing. Multifunctional epoxy chain extenders have been reported to react with terminal hydroxyl and carboxyl groups of polyesters, potentially leading to chain extension and, depending on functionality and reaction extent, branched molecular structures.^79,80^ However, FTIR does not directly resolve changes in molecular architecture and, therefore, cannot independently confirm chain extension or long-chain branching in the present system. Accordingly, the FTIR observations are interpreted as supporting evidence for reactive interactions rather than direct proof of the proposed reaction mechanism.

### Mechanical Characterization

The tensile behavior of neat PHB, PBAT, PHB/PBAT blends, and PHB/PBAT/Joncryl/MgO nanocomposites was evaluated from the representative stress-strain curves shown in Figure 3a,b, while the corresponding tensile and impact properties are summarized in Table 2.

**Figure 3:**
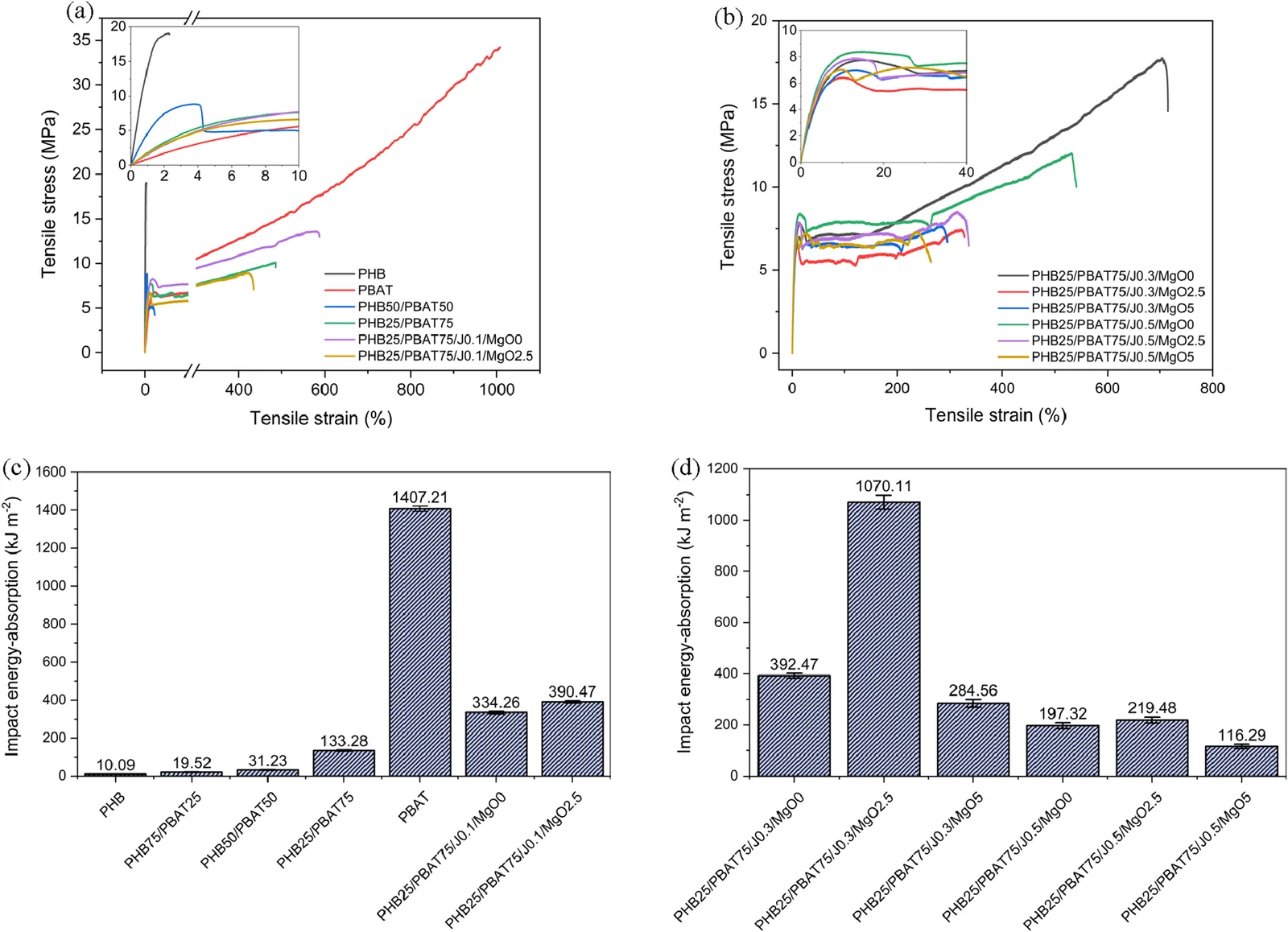
Mechanical behavior of PHB, PBAT, PHB/PBAT blends, and PHB/PBAT/Joncryl/MgO nanocomposites: representative tensile stress-strain curves of (a) neat PHB, PBAT, and PHB/PBAT blends with different blend ratios, and (b) compatibilized PHB/PBAT/Joncryl/MgO nanocomposites with varying Joncryl and MgO contents; impact-absorbed energy of (c) neat PHB, PBAT, and PHB/PBAT blends, and (d) compatibilized PHB/PBAT/Joncryl/MgO nanocomposites with different Joncryl and MgO loadings. Impact-absorbed energy values in (c) and (d) are presented as mean ± standard deviation from at least five independent specimens per formulation. Stress-strain curves in (a) and (b) are representative individual curves.

**Table 2:** Mechanical Properties of PHB/PBAT/MgO Nanocomposite[Table-fn t2fn1]

sample	ultimate tensile stress (MPa)	Young’s modulus (MPa)	tensile strain (%)	yield stress (MPa)	toughness (MPa) or (MJ m^–3^)
PHB	19.04 ± 1.6^b^	1623 ± 13^a^	2 ± 0.2^g^	19.01 ± 1.6^a^	0.31 ± 0.21^g^
PHB/PBAT 50:50	9.16 ± 0.9^de^	491 ± 9^b^	21 ± 3.2^g^	8.82 ± 0.9^b^	1.15 ± 0.17^g^
PHB/PBAT 25:75	9.54 ± 1.1^de^	215 ± 4^c^	485 ± 11.8^cd^	7.73 ± 1.1^bcd^	36.37 ± 1.7^d^
PBAT	34.21 ± 0.5^a^	36 ± 2^c^	1062 ± 15.3^a^	6.77 ± 0.2^cd^	169.64 ± 12^a^
PHB/PBAT/J 25:75:0.1	13.16 ± 0.5^c^	155 ± 3^c^	579 ± 8.3^bc^	8.29 ± 0.3^bc^	57.24 ± 6.9^c^
PHB/PBAT/J/MgO 25:75:0.1:2.5	8.39 ± 0.8^e^	173 ± 5^c^	416 ± 7.9^cde^	6.59 ± 0.8^cd^	29.34 ± 5.5^de^
PHB/PBAT/J/MgO 25:75:0.1:5	8.94 ± 1.1^de^	184 ± 6^c^	324 ± 7.5^def^	6.86 ± 1.1^cd^	23.05 ± 8.4^ef^
PHB/PBAT/J 25:75:0.3	17.73 ± 0.6^b^	137 ± 5^c^	705 ± 10.7^b^	7.79 ± 0.6^bcd^	78.66 ± 6.9^b^
PHB/PBAT/J/MgO 25:75:0.3:2.5	7.45 ± 0.9^e^	154 ± 7^c^	322 ± 6.9^def^	6.40 ± 0.9^d^	19.75 ± 4.7^f^
PHB/PBAT/J/MgO 25:75:0.3:5	7.67 ± 0.9^e^	176 ± 6^c^	284 ± 5.1^ef^	6.96 ± 0.9^bcd^	19.59 ± 4.9^f^
PHB/PBAT/J 25:75:0.5	12.03 ± 0.6^cd^	197 ± 6^c^	531 ± 8.1^c^	8.32 ± 0.6^bc^	48.46 ± 5.3^c^
PHB/PBAT/J/MgO 25:75:0.5:2.5	8.51 ± 1.1^e^	190 ± 8^c^	315 ± 7.9^def^	7.85 ± 1.1^bcd^	24.15 ± 8.5^ef^
PHB/PBAT/J/MgO 25:75:0.5:5	7.31 ± 1.3^e^	187 ± 9^c^	237 ± 6.8^f^	7.04 ± 1.3^cd^	17.36 ± 8.7^f^

* Values are reported as mean ± standard deviation (*n* = 5). Different superscript letters within the same column indicate statistically significant differences among formulations based on one-way ANOVA followed by Tukey’s HSD post-hoc test (*p* < 0.05).

As shown in Figure 3a, neat PHB exhibits a steep initial slope followed by fracture at a strain of approximately 2%, confirming its brittle behavior and limited energy absorption. In contrast, PBAT undergoes extensive plastic deformation with pronounced strain hardening, resulting in the highest toughness among the investigated materials. Increasing the PBAT content progressively shifted the mechanical response from brittle to ductile behavior. The PHB/PBAT (25:75) blend exhibited a substantial increase in elongation at break and toughness compared to PHB-rich blends, indicating that deformation was increasingly governed by the continuous PBAT phase. The resulting increase in plastic deformation is consistent with more effective energy dissipation before fracture.

On the basis of these results, the PHB/PBAT (25:75) blend was selected as the reference composition for subsequent investigations because it provided the best combination of ductility, melt processability, filament continuity, and FDM printability while maintaining adequate mechanical integrity. Using a single reference composition also enabled the effects of reactive compatibilization and MgO incorporation to be evaluated without the additional influence of the blend composition.

Reactive compatibilization further improved the tensile response of the PHB/PBAT (25:75) blend. As shown in Figure 3b, Joncryl increased the stress sustained over a broader strain range, resulting in a larger area under the stress-strain curve. These observations agree with the tensile properties summarized in Table 2, which show higher tensile strength and toughness for the compatibilized blends than for the uncompatibilized blend.

The pronounced increase in elongation at break after addition of 0.3 wt % Joncryl suggests that reactive modification altered the deformation behavior of the PHB/PBAT blend, allowing the material to sustain substantially larger strains before failure. This behavior is examined further in the morphology section, where the fracture surface features are considered in relation to interfacial continuity and deformation behavior.

The reduction in toughness at higher Joncryl contents is unlikely to result from solely restricted chain mobility. Excessive reactive modification may also alter the phase morphology and crystallization behavior, reducing the effectiveness of energy-dissipation mechanisms during fracture. The superior mechanical performance obtained with 0.3 wt % Joncryl therefore appears to reflect an optimal balance among melt rheology, morphology development, and crystallization behavior rather than an increasing degree of reactive compatibilization alone.

The influence of MgO is shown in Figure 3b. At a fixed Joncryl content, increasing the MgO loading produced shorter stress-strain curves with lower elongation and a smaller area under the curve. This trend was consistent across all Joncryl concentrations and is reflected quantitatively in Table 2, where both elongation at break and toughness decreased as MgO content increased. These results indicate that MgO reduces the ductility and limits energy absorption. Although Young’s modulus increased slightly in some MgO-containing formulations, the overall mechanical response shifted toward higher stiffness and lower ductility. This behavior is consistent with restricted polymer chain mobility and localized stress concentrations associated with the presence of rigid inorganic particles. However, the present results do not allow a specific particle-scale mechanism to be assigned to the observed decrease in ductility. These results indicate that MgO incorporation involves a mechanical performance penalty, particularly at higher loadings; the significance of this trade-off relative to the degradation-related functionality of MgO is discussed in the degradation section.

The impact-absorbed energy results (Figure 3c,d) followed trends similar to those observed in tensile testing. The uncompatibilized PHB/PBAT blend exhibited relatively low impact resistance because of its coarse phase-separated morphology and weak interfacial interactions promoted premature crack initiation and propagation. Reactive compatibilization substantially increased the absorbed impact energy, indicating an improved resistance to fracture. SEM revealed a finer dispersed-phase morphology after reactive compatibilization, while DSC showed the corresponding changes in crystallization behavior. Together, these observations are consistent with the enhanced impact performance of compatibilized blends. Among the investigated formulations, PHB/PBAT/J0.3 exhibited the highest impact resistance, suggesting that moderate reactive modification provided the most favorable balance between the stiffness and toughness. Although the individual contributions of morphology, crystallization behavior, and melt rheology cannot be isolated from the present results, the combined evidence indicates that these factors collectively contributed to the superior impact performance of this formulation.^41–43^

Moderate MgO loadings maintained an acceptable balance between stiffness and toughness, whereas higher MgO contents reduced impact resistance, indicating that increasing the filler content restricted the ability of the material to dissipate energy during fracture. This behavior is consistent with the accompanying reduction in ductility, although the present results do not identify a specific particle-scale mechanism responsible for this change.

The tensile and impact results demonstrate that reactive compatibilization effectively mitigated the stiffness-toughness trade-off of the PHB/PBAT blends. The results also show that moderate Joncryl loading provided the most favorable mechanical response, whereas MgO shifted the behavior toward a higher stiffness and lower ductility.

### Thermal Analysis

Thermogravimetric analysis (TGA) was conducted to evaluate the thermal degradation behavior of PHB, PBAT, and PHB/PBAT blends and PHB/PBAT/MgO nanocomposites with varying Joncryl and MgO contents (Figure 4a,b).

**Figure 4:**
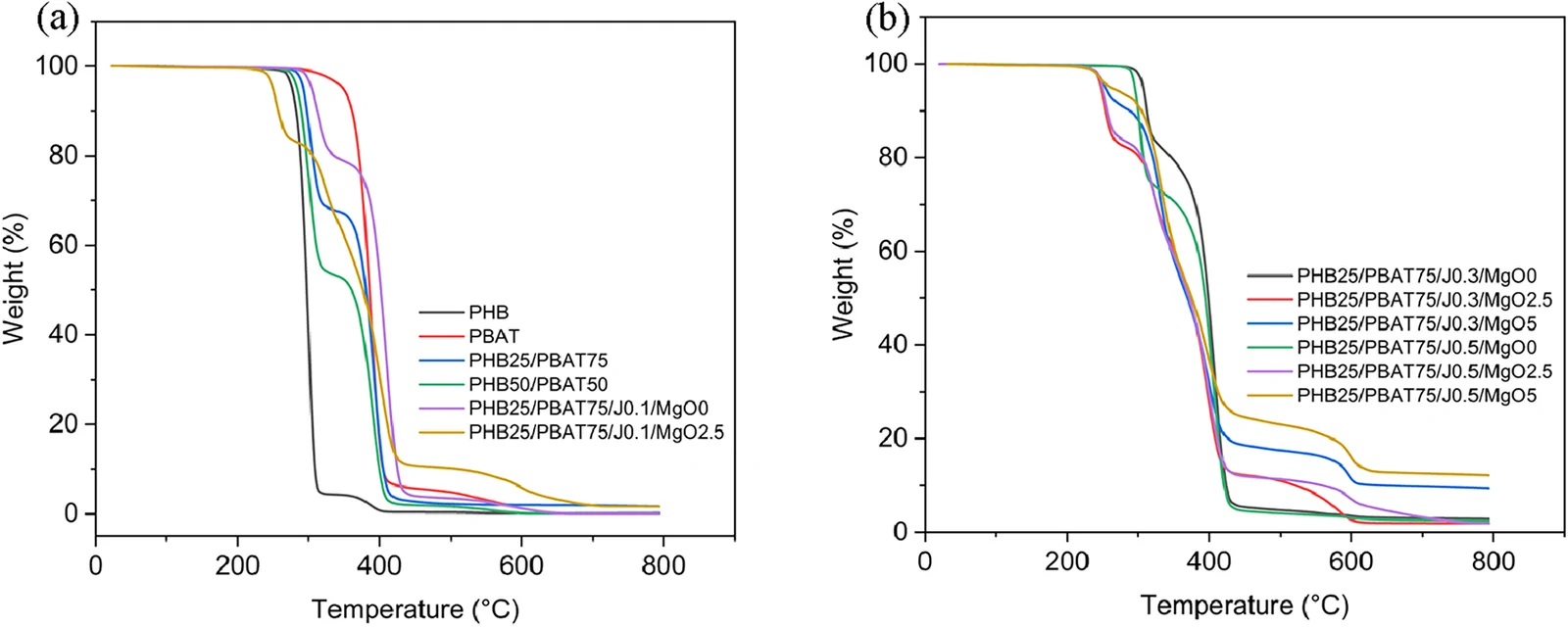
TGA curves of (a) neat PHB, PBAT, and PHB/PBAT blends with different blend ratios, and (b) compatibilized PHB/PBAT/Joncryl/MgO nanocomposites with varying Joncryl and MgO contents under a nitrogen atmosphere.

The TGA curves (Figure 4a) show that neat PHB undergoes rapid single-step degradation between approximately 280 and 320 °C, whereas PBAT degrades at higher temperatures (approximately 350–420 °C), reflecting its greater thermal stability. PHB/PBAT blends exhibit a two-step degradation profile corresponding to the independent decomposition of PHB and PBAT phases, consistent with the immiscible nature of blends.

The addition of Joncryl shifted the onset of degradation to higher temperatures compared to the uncompatibilized PHB/PBAT blend, indicating improved thermal stability. This shift is consistent with previous reports on epoxy-functional chain extenders in polyester systems, where reactions with terminal hydroxyl and carboxyl groups can reduce the concentration of thermally labile chain ends and increase effective molecular connectivity through chain extension and possible branching.^81–83^ In the present system, the TGA results are therefore consistent with but do not independently confirm such changes in molecular architecture.

In contrast, MgO nanoparticles shifted the onset of degradation to lower temperatures (Table S1), with the effect becoming more pronounced as the MgO content increased. Under the dry nitrogen atmosphere used for TGA, MgO did not improve thermal stability but instead promoted earlier thermal decomposition (Figure 4b). This behavior is consistent with catalytic interactions between MgO surfaces and polyester ester bonds, which may accelerate the thermally induced chain scission. These observations should not be directly compared to hydrolytic degradation, as the mechanisms governing thermal decomposition under inert conditions differ fundamentally from those that operate in aqueous environments.

The DTGA curves (Figure S2a,b) further support the TGA results. Neat PHB and PBAT exhibited distinct degradation peaks centered near 300 and 400 °C, respectively, whereas the blends showed two characteristic peaks associated with the degradation of each polymer phase. Reactive compatibilization broadened these peaks and increased their overlap, consistent with enhanced interactions between the two polymer phases. Increasing the MgO content shifted the DTGA peaks toward lower temperatures, confirming that MgO accelerated thermal degradation under the applied TGA conditions.

The TGA and DTGA results indicate that reactive compatibilization improved the thermal stability of the PHB/PBAT blends, whereas MgO promoted an earlier thermal decomposition under inert conditions. These findings emphasize that the role of MgO depends on the degradation environment and should therefore be distinguished from its behavior during aqueous degradation.

Figure 5a–d present the DSC cooling and heating curves of PHB, PBAT, PHB/PBAT blends, and PHB/PBAT/MgO nanocomposites, illustrating the effects of blend composition, reactive compatibilization, and MgO incorporation on crystallization and melting behavior.

**Figure 5:**
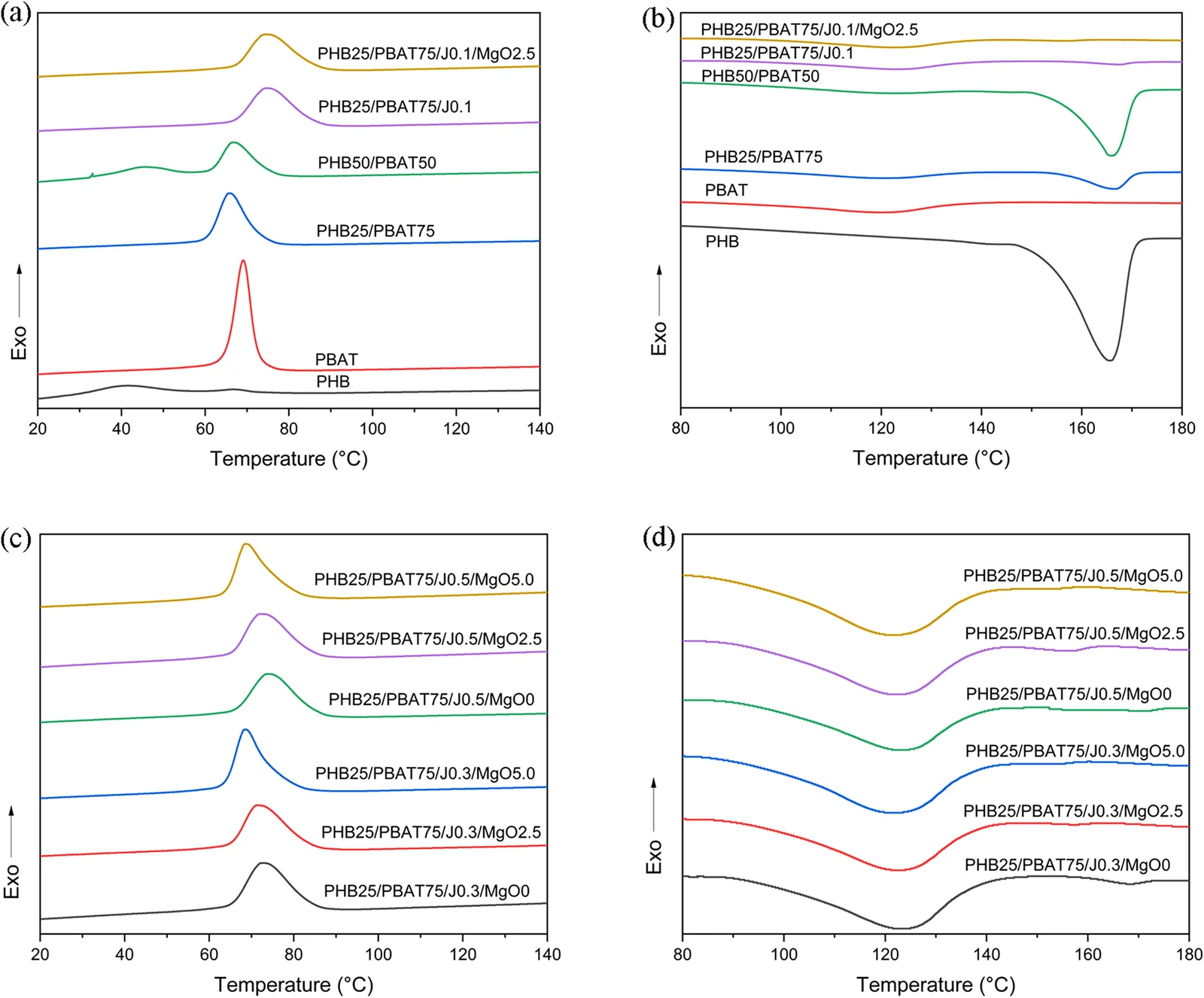
DSC curves of PHB, PBAT, PHB/PBAT blends, and PHB/PBAT/MgO nanocomposites: cooling curves of (a) neat PHB, PBAT, and PHB/PBAT blends with different blend ratios, and (c) compatibilized PHB/PBAT/MgO nanocomposites with varying Joncryl and MgO contents; second heating curves of (b) neat PHB, PBAT, and PHB/PBAT blends, and (d) compatibilized PHB/PBAT/MgO nanocomposites.

During cooling (Figure 5a), neat PHB and PBAT exhibit distinct crystallization behaviors. Both crystallization peaks are evident in the blends at intermediate compositions; however, the PHB crystallization peak becomes progressively weaker as the PHB content decreases. In the PHB/PBAT (25:75) blend, the PHB crystallization peak is nearly absent, indicating that the reduced continuity of the PHB-rich phase limits the formation of well-developed PHB crystals.

Reactive compatibilization shifted the PHB crystallization temperature (*T*_c_) to higher values while substantially reducing the crystallization enthalpy (Δ*H*_c_). These results indicate that crystallization began earlier during cooling but proceeded to a lower overall degree of crystallization, consistent with modified crystallization kinetics following reactive processing.

MgO produced the opposite effect. Compared with the Joncryl-only blend, MgO reduced the crystallization peak area and shifted the *T*_c_ to lower temperatures, suggesting delayed crystallization and lower crystallization efficiency. In the compatibilized PBAT-rich systems (Figure 5c), no distinct PHB crystallization peak was observed, confirming that crystallization was dominated by the PBAT phase.

At a fixed MgO content, increasing the Joncryl concentration shifted the PBAT crystallization peak to higher temperatures, whereas increasing the MgO content at a fixed Joncryl concentration shifted the peak to lower temperatures. No consistent trend was observed in the crystallization peak area with an increasing MgO loading.

The heating curves (Figure 5b,d) provide complementary information on the melting behavior. Neat PHB exhibited a sharp melting peak, whereas PBAT showed a broad melting transition. As the PBAT content increased, the PHB melting peak became progressively less pronounced. Reactive compatibilization further suppressed the PHB melting peak, whereas the PBAT melting behavior remained largely unchanged. MgO primarily affects the PHB phase. A weak PHB melting peak remained detectable in the compatibilized blend without MgO but gradually disappeared with an increase in MgO loading. This behavior suggests that MgO further modified PHB crystallization, whereas its influence on the PBAT crystalline structure was comparatively limited under the present conditions.

The degrees of crystallinity of the PBAT and PHB phases were estimated separately by normalizing the corresponding melting enthalpies to those of the fully crystalline polymers. For each phase, crystallinity was calculated as *X*_C_ = (Δ*H*_m_–Δ*H*_cc_) / *(*Δ*H*_m_^*^ × *W*), where Δ*H*_m_ is the melting enthalpy obtained from DSC analysis, Δ*H*_cc_ is the cold crystallization enthalpy (negligible in our samples), Δ*H*_m_^*^ is the melting enthalpy of a 100% crystalline polymer, which equals 114 J g^–1^ for PBAT^84^ and 146 J g^–1^ for PHB,^85^ and *W* is the mass fraction of the corresponding polymer in the formulation. The calculated crystallinity values are summarized in Table S1. PHB crystallinity decreased markedly with increasing PBAT content and was strongly suppressed after reactive modification, consistent with the reduced PHB melting and crystallization features observed in the DSC thermograms. For formulations in which the PHB melting transition could not be reliably resolved, PHB crystallinity was not calculated.

The DSC results show that reactive compatibilization and MgO incorporation influenced the crystallization behavior of the PHB/PBAT blends in different ways. Joncryl promoted earlier crystallization while reducing the overall degree of crystallinity, whereas MgO delayed crystallization and further suppressed the PHB crystal development. These changes provide additional insight into the differences in the mechanical performance observed among the investigated formulations.

### Rheological Characterization and Melt Processing Behavior

The rheological response (Figures 6 and 7) provides a direct measure of melt viscoelasticity during processing and serves as the primary framework for interpreting the processing behavior of the investigated system. In immiscible polymer blends, rheological properties reflect not only changes in the molecular architecture associated with reactive processing but also the evolution of the dispersed-phase morphology during melt processing. Consequently, variations in storage modulus, complex viscosity, and relaxation behavior should be interpreted as indicators of the coupled effects of molecular modification, viscosity ratio, droplet deformation, and coalescence rather than as evidence of reactive compatibilization alone.

**Figure 6:**
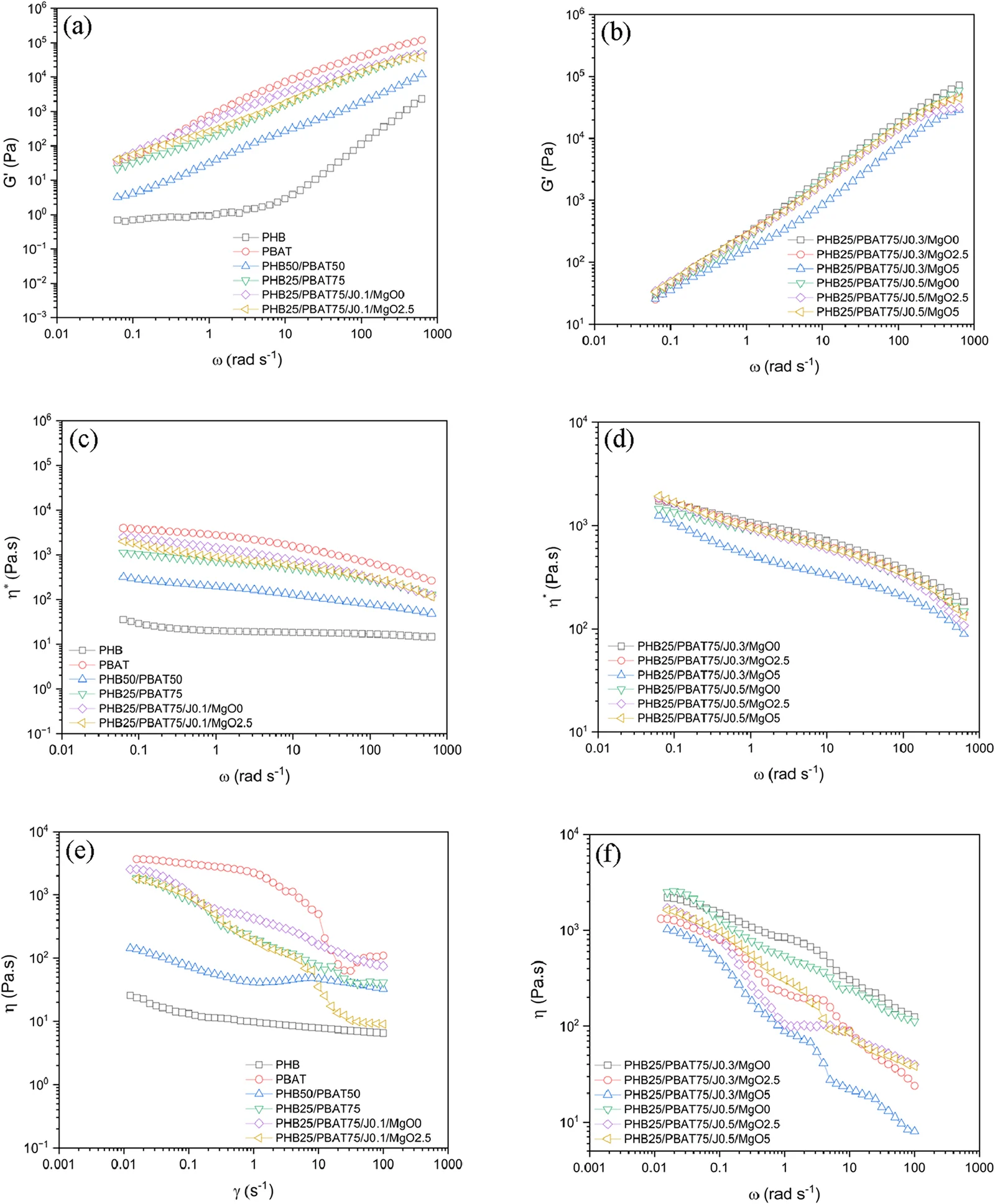
Rheological behavior of PHB, PBAT, PHB/PBAT blends, and PHB/PBAT/MgO nanocomposites: frequency dependence of (a, b) storage modulus (*G*′), (c, d) complex viscosity (η*), (e, f) shear viscosity (η).

**Figure 7:**
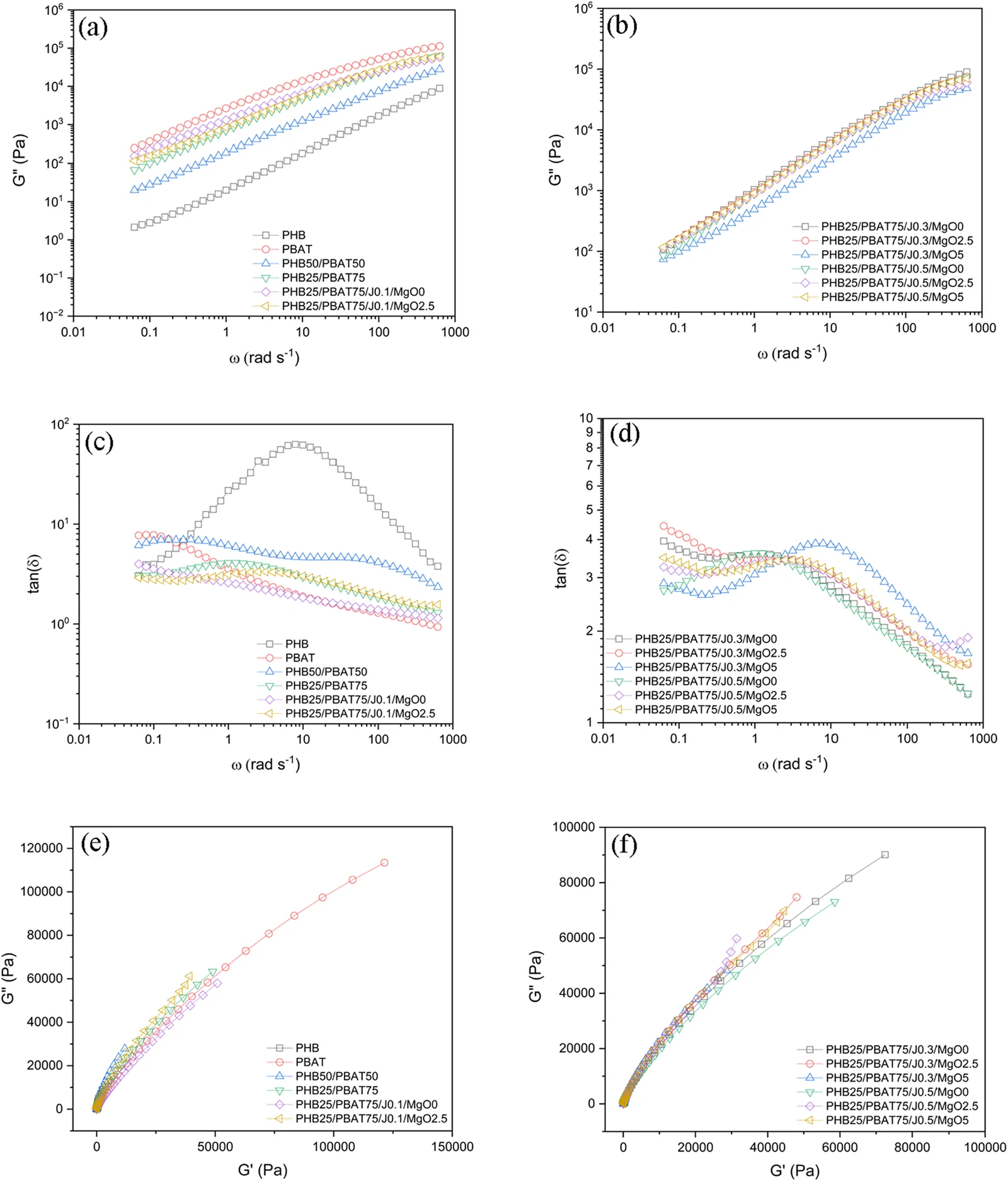
Rheological behavior of PHB, PBAT, PHB/PBAT blends, and PHB/PBAT/MgO nanocomposites: frequency dependence of (a, b) loss modulus (*G*″), (c, d) tanδ, and (e, f) Cole–Cole plots.

The storage modulus (*G*′) as a function of angular frequency exhibits a strong dependence on composition and reactive modification. Neat PHB and PBAT show distinctly different *G*′ responses, reflecting differences in their molecular structure and melt relaxation behavior. Because PHB and PBAT are chemically distinct polymers, the difference in *G*′ between the two neat polymers cannot be used directly to infer differences in their degree of chain entanglement. Notably, neat PHB exhibits a low-to-intermediate-frequency plateau-like response rather than simple terminal behavior, suggesting the presence of slowly relaxing structural constraints in the melt. Such behavior could be associated with slowly relaxing structures generated during thermal processing, including possible branching or partial network formation; however, the present measurements do not allow the molecular origin of this response to be identified. The PHB/PBAT blends exhibit intermediate and composition-dependent behavior arising from contributions of both phases and their multiphase morphology.

Upon incorporation of Joncryl, a pronounced increase in *G*′ is observed across the entire frequency range, particularly in the low-frequency region, indicating enhanced melt elasticity, consistent with changes in molecular architecture from epoxy-mediated reactive processing.

The increase in the storage modulus should also be considered from the perspective of morphology development during reactive melt blending. In immiscible polymer blends, the deformation and breakup of dispersed domains are strongly influenced by the viscosity ratio between the dispersed and the continuous phases. Epoxy-mediated reactive processing can increase the viscosity and elasticity of the blend, thereby modifying the viscosity ratio and reducing the tendency of the dispersed domains to undergo coalescence during extrusion. As a result, the rheological response reflects not only molecular-level changes but also the evolution of the blend morphology during the processing. The finer dispersed morphology observed by SEM in the compatibilized blends (Figure 9e–g) is consistent with this interpretation. However, the transient evolution of the droplet breakup and coalescence was not monitored directly. Accordingly, the proposed relationship between the rheology and morphology should be regarded as an interpretation rather than direct experimental evidence.

To provide a more physically meaningful interpretation of the rheological response, a characteristic relaxation time (λ) can be estimated from the ratio of complex viscosity to storage modulus (λ ≈ η*/*G*′ at low frequency). This parameter reflects the time scale over which the polymer melt relaxes under applied stress and provides insight into the balance between flowability and structural recovery during processing. In the context of FDM processing, this time scale plays a critical role. If λ is too small, the material relaxes rapidly after extrusion, leading to poor filament shape retention and increased defect formation. Conversely, excessively large λ values can hinder flow and reduce interlayer fusion. Therefore, an intermediate relaxation regime is required to achieve stable filament formation and adequate interlayer bonding.

Based on the relative changes in η* and *G*′, the effective relaxation time is expected to increase with reactive compatibilization due to enhanced chain connectivity and longer relaxation modes. In contrast, MgO incorporation modifies the relaxation behavior by introducing rigid particulate heterogeneity that locally restricts polymer-chain mobility and alters stress redistribution within the melt.

A comparison of Joncryl content shows a non-monotonic effect on the viscoelastic behavior of the blends. As shown in Figure 6b, the formulation containing 0.3 wt % Joncryl exhibits somewhat higher G′ values than the corresponding 0.1 wt % formulation over portions of the measured frequency range. Increasing the Joncryl content further to 0.5 wt % does not produce an additional increase in G′. This non-monotonic response suggests that the effect of Joncryl concentration on melt elasticity is not simply proportional to its loading. The observed trend may reflect differences in the extent of chain extension and branching during reactive processing; however, the molecular architecture was not directly characterized in this study. This behavior suggests that an optimum compatibilizer concentration exists for the present PHB/PBAT system. Similar non-monotonic rheological responses have been reported for multifunctional epoxy chain extenders, where excessive epoxy functionality may alter the balance between chain extension, branching, and network homogeneity, leading to less efficient stress transmission through the transient viscoelastic network. Since the molecular architecture was not directly characterized in the present study, this interpretation should be regarded as a plausible explanation rather than direct experimental proof. Therefore, 0.3 wt % Joncryl appears to provide the most favorable balance between reactive modification and melt viscoelasticity.

The frequency dependence of *G*′ provides additional insight into melt response. As shown in Figure 6a, neat PHB exhibits a relatively frequency-independent *G*′ over the low-to-intermediate-frequency range, followed by a more pronounced increase at higher frequencies. This plateau-like response is inconsistent with simple terminal flow and indicates the presence of slowly relaxing structural constraints in the PHB melt. Such behavior may arise from processing-induced changes in molecular architecture, including branching or partial network formation, although the present rheological data do not establish the molecular origin of these constraints.

The loss modulus (*G*″) increased after reactive compatibilization. Because the increase in *G*′ exceeded that of *G*″, tanδ decreased, indicating a shift toward more elastic melt behavior with a longer relaxation mode. Reactive compatibilization also increased the complex viscosity, particularly at low frequencies, indicating greater resistance to flow under quiescent conditions. The maximum η* was obtained for the formulation containing 0.3 wt % Joncryl, whereas a slight decrease was observed at 0.5 wt %, suggesting an optimum degree of reactive modification for the present system.

The incorporation of MgO produces a distinct decrease in *G*′ and η*, particularly in the low-frequency region (Figure 6b,d). This behavior cannot be explained by a simple filler-volume effect, which would generally be expected to increase resistance to flow. Moreover, the decrease cannot be directly attributed to particle agglomeration because the present SEM analysis does not provide quantitative evidence of MgO agglomerate size or distribution. Instead, the observed decrease in *G*′ and η* indicates an apparent plasticization-like rheological response. One possible explanation is that MgO alters the reactive environment during melt processing and thereby affects the extent of Joncryl-mediated chain extension and branching, resulting in lower effective molecular connectivity. Changes in local chain packing and interfacial mobility may also contribute to the observed response. However, the present results do not allow these contributions to be separated or a single mechanism to be assigned.

All systems exhibit pronounced shear thinning behavior, which becomes more evident in compatibilized systems. The enhanced shear thinning behavior reflects progressive molecular orientation and relaxation under increasing deformation, which reduces flow resistance at higher shear rates while maintaining greater melt resistance at low shear rates. This combination of high low-shear viscosity and strong shear-thinning behavior is particularly advantageous for fused deposition modeling (FDM) as it enables stable filament formation while allowing smooth extrusion through the nozzle.

To further relate rheological behavior to FDM processing performance, a dimensionless rheological printability index (RPI) was defined based on low-frequency viscoelastic properties:

$$\mathrm{RPI}=\left(\eta^{*}/{\eta_{\mathrm{ref}}}^{*}\right)/\left(\mathrm{tan\delta}/{\mathrm{tan\delta}}_{\mathrm{ref}}\right)\;\left(\mathrm{evaluated}\;\mathrm{at}\;1\;\mathrm{rad}\;s^{-1}\right)$$

where η* and tanδ are the complex viscosity and loss factor of each formulation, respectively, and *η*_ref_^*^ and tanδ_ref_ correspond to the uncompatibilized PHB/PBAT 25:75 reference blend measured at the same angular frequency. The RPI was introduced here as a semi-quantitative comparative descriptor rather than as an independently validated predictor of FDM printability. Its formulation combines two rheological characteristics that are relevant to filament extrusion and post-deposition stability. Complex viscosity reflects the resistance of the melt to deformation under low-frequency conditions, whereas tanδ (=*G*″/*G*′) describes the relative contribution of viscous and elastic responses. Normalization to the uncompatibilized PHB/PBAT 25:75 blend provides a dimensionless basis for comparing the investigated formulations under the same processing framework. Accordingly, an increase in η* together with a decrease in tanδ produces a higher RPI, representing greater low-frequency melt resistance combined with a more elastic response. Such a rheological balance can favor filament integrity and post-deposition shape retention, provided that the viscosity remains sufficiently low to permit stable flow through the printing nozzle. Within the formulation space investigated here, RPI values greater than 1 therefore indicate a rheological balance that is potentially more favorable for filament stability and shape retention relative to the reference blend, whereas values below 1 indicate a less favorable balance of low-frequency viscous resistance and elastic response. The RPI should not, however, be interpreted as a universal or monotonically predictive measure of printability. In the present study, its relevance is limited to semi-quantitative comparison among the investigated formulations, and the observed relationship between RPI and FDM behavior is qualitative rather than an experimentally validated quantitative correlation. Rigorous validation of this parameter would require quantitative print-quality metrics over a broader formulation and processing space and statistical correlation of those metrics with RPI. Representative rheological parameters and corresponding RPI values are summarized in Table 3.

**Table 3:** Representative Rheological Parameters of Selected PHB/PBAT/MgO Nanocomposites

sample	*G*′ (Pa) @ 1 rad s^–1^	η* (Pa·s) @ 1 rad s^–1^	tanδ @ 1 rad s^–1^	RPI	shear-thinning behavior
PHB	0.913	19.93	21.71	0.005	weak
PHB/PBAT 25:75	170.15	719.11	4.09	1.000	moderate
PHB/PBAT/J 25:75:0.3	285.11	1076.9	3.59	1.706	strong
PHB/PBAT/J/MgO 25:75:0.3:5	159.23	516.48	3.07	0.957	strong

The RPI values provide a semiquantitative perspective on the interplay between viscous resistance and elastic response within the formulation space investigated here. Neat PHB exhibits a very low RPI, reflecting its low complex viscosity and predominantly viscous response at 1 rad s^–1^. Blending with PBAT substantially increases the RPI, indicating a more balanced low-frequency viscoelastic response relative to that of neat PHB and the reference blend.

The PHB/PBAT/J0.3 formulation exhibits the highest RPI, resulting from the combined increase in complex viscosity and decrease in tanδ. This combination indicates greater resistance to low-frequency deformation together with a stronger elastic contribution to the melt response. Such behavior is potentially favorable for filament integrity and post-deposition shape retention, provided that the melt remains sufficiently flowable for stable extrusion through the nozzle.

In contrast, incorporation of MgO reduces the RPI, primarily because of the decrease in complex viscosity despite the lower tanδ. This result suggests that MgO alters the balance between viscous resistance and elastic response and is consistent with the changes observed in the frequency-dependent rheological behavior. Within the present formulation set, the lower RPI of the MgO-containing system was qualitatively associated with less favorable filament-forming behavior compared with the Joncryl-modified formulation without MgO. However, because quantitative print-quality metrics were not independently correlated with RPI, these observations should not be interpreted as experimental validation of a direct relationship between RPI and FDM printability. Rather, the RPI is used here as a semi-quantitative descriptor for comparing the viscoelastic balance of the investigated formulations under the same processing framework.

The Cole–Cole plots provide further insight into the relaxation behavior of the system. The uncompatibilized blends exhibit deviations from ideal semicircular behavior, indicating a broad and heterogeneous relaxation spectrum associated with phase separation. The addition of Joncryl results in smoother and more continuous curves, suggesting a narrower relaxation time distribution and an improved interfacial compatibility. In contrast, MgO-containing systems show renewed deviations from ideal behavior, indicating increased heterogeneity and the presence of multiple relaxation mechanisms arising from polymer–filler interactions.

Stress relaxation measurements were further performed to directly evaluate the evolution of melt relaxation dynamics and network behavior in the developed systems. Figure 8a,b shows the relaxation modulus *G*(*t*) as a function of time for neat polymers, blends, and compatibilized nanocomposites.

**Figure 8:**
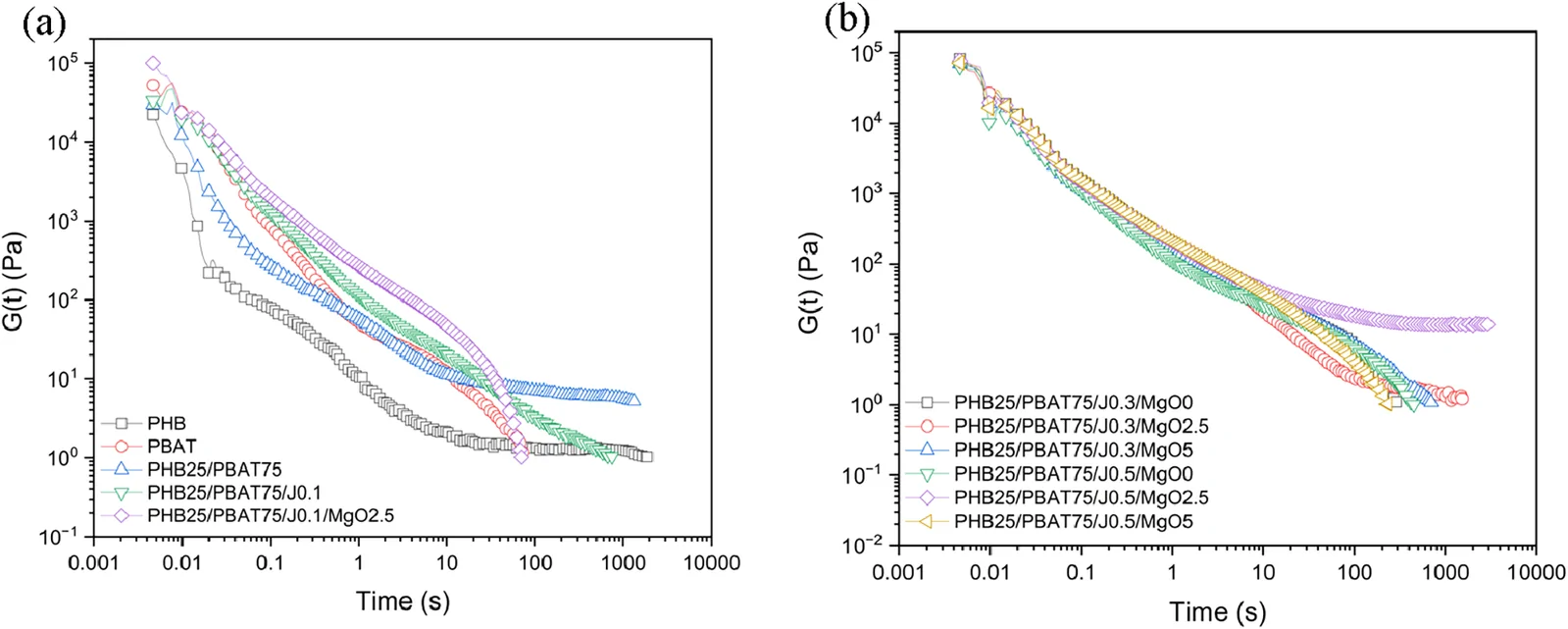
Stress relaxation behavior of PHB, PBAT, PHB/PBAT blends, and PHB/PBAT/MgO nanocomposites: relaxation modulus (*G*(*t*)) as a function of time for (a) neat PHB, PBAT, and PHB/PBAT blends with different blend ratios, and (b) compatibilized PHB/PBAT/Joncryl/MgO nanocomposites with varying Joncryl and MgO contents.

Neat PHB exhibits relatively rapid stress dissipation over much of the measured time range, whereas PBAT shows a slower relaxation response. These differences reflect the distinct molecular structures and relaxation dynamics of the two polymers and should not be interpreted simply in terms of differences in the chain entanglement.

Reactive compatibilization with Joncryl produces a clear change in the relaxation response. Compared with uncompatibilized blends, Joncryl-containing systems exhibit a slower decay of *G*(*t*), indicating slower relaxation stress and a broader distribution of relaxation processes.

The incorporation of MgO nanoparticles further modifies the relaxation behavior through polymer–filler interactions and localized chain confinement. Extended relaxation tails observed at longer times suggest heterogeneous relaxation mechanisms and delayed stress-dissipation processes.

From a processing perspective, slower stress relaxation improves melt shape retention after extrusion and enhances the filament stability during FDM processing. However, excessively delayed relaxation may hinder polymer chain diffusion across deposited layers and reduce the interlayer bonding efficiency. Therefore, an intermediate relaxation regime appears to be desirable to balance dimensional stability and interlayer adhesion.

Overall, reactive compatibilization produced a more elastic melt characterized by higher storage modulus, higher complex viscosity, lower tanδ, and longer relaxation times. These changes explain the improved filament stability and FDM processability observed for the compatibilized formulations and demonstrate the value of rheological characterization as a framework for understanding processing-structure relationships in biodegradable PHB/PBAT systems. Although several mechanistic interpretations are consistent with previous studies, the individual contributions of chain extension, branching, and interfacial compatibilization could not be isolated experimentally and therefore warrant further investigation.

### Morphology

Field-emission scanning electron microscopy (FESEM) was used to examine the fracture morphology, phase dispersion, and interfacial characteristics of PHB/PBAT blends and PHB/PBAT/MgO nanocomposites. To facilitate interpretation, key microstructural features, including voids, cracks, particle pullout, and interfacial gaps, are highlighted in the corresponding micrographs.

Figure 9a–d show the fracture surfaces of neat PHB, PBAT, and the uncompatibilized PHB/PBAT blends. The blends exhibit heterogeneous morphologies with clear phase separation, indicating poor interfacial adhesion between the PHB and the PBAT phases. Voids and interfacial gaps are frequently observed and likely act as stress-concentration sites during deformation. These morphological features are consistent with the relatively low elongation at break and impact resistance of the incompatible blends.

**Figure 9:**
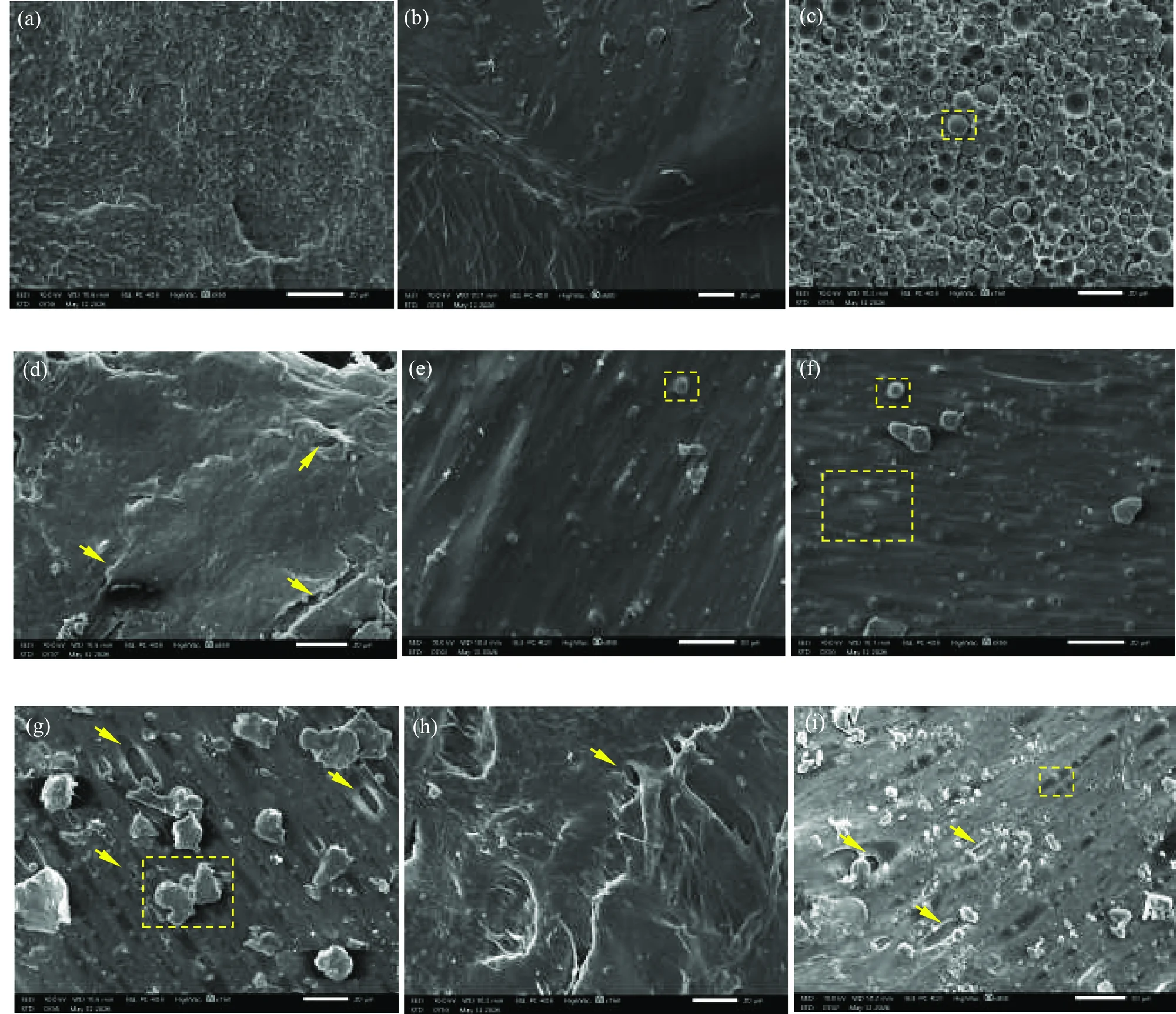
SEM micrographs of fractured surfaces of PHB, PBAT, PHB/PBAT blends, and PHB/PBAT/Joncryl/MgO nanocomposites: (a–d) neat PHB, neat PBAT, PHB75/PBAT25, and PHB25/PBAT75 blends, respectively, and (e–g) compatibilized PHB25/PBAT75 blends with varying Joncryl (0.1, 0.3, and 0.5, respectively), and (h, i) compatibilized PHB25/PBAT75/J0.3 with varying MgO contents (2.5 and 5 wt %, respectively), illustrating changes in phase morphology, interfacial adhesion, and polymer–filler interactions. The yellow arrows indicate representative morphological features discussed in the text. The yellow dashed squares identify representative dispersed PHB domains within the continuous PBAT-rich matrix, highlighting the evolution of dispersed-phase morphology following reactive compatibilization and MgO incorporation.

The effect of reactive compatibilization is evident in Figure 9e–g. The interfaces between the PHB and PBAT phases become less distinct, and the fracture surfaces appear to be more homogeneous. The reduction in interfacial gaps and the more uniform morphology are consistent with an improved compatibility between the two polymer phases. These morphological changes help explain the large increase in the elongation at break observed for the compatibilized blends. Following reactive modification, the more homogeneous fracture morphology and reduced interfacial discontinuities are consistent with more effective stress transfer between the PHB- and PBAT-rich regions. This condition allows the continuous PBAT-rich phase to undergo more extensive plastic deformation before fracture. The particularly high elongation observed for the PHB/PBAT/J0.3 formulation therefore appears to reflect a favorable balance between improved interfacial continuity and the retention of PBAT-dominated chain mobility. At higher Joncryl content, this balance may be partially lost, consistent with the reduction in elongation and toughness observed for the 0.5 wt % formulation.

These morphological changes are consistent with the reactive processing mechanism proposed in Figure 1. Although the SEM observations do not directly reveal the underlying chemical reactions or interfacial bonding mechanisms, they support the interpretation that reactive compatibilization influenced the phase development during melt processing.

In the compatibilized PHB/PBAT/MgO nanocomposites (Figure 9h,i), MgO particles are visible throughout the examined fracture surfaces. However, the present SEM images do not provide sufficient resolution or sampling to quantitatively assess particle dispersion or agglomerate size distribution. Therefore, no conclusion regarding the degree of MgO dispersion or agglomeration can be drawn from these images. The fracture surfaces nevertheless show that the addition of MgO modifies the local morphology of the compatibilized blend, although the relationship between these morphological features and the rheological response cannot be established from the SEM alone. Accordingly, the micrographs are used here only to describe the observed morphology and the presence of MgO-rich features within the polymer matrix.

The compatibilized blends exhibited fracture surfaces characteristic of more ductile deformation, whereas the uncompatibilized blends showed brittle fracture features including clean cracks, voids, and interfacial debonding. The SEM observations therefore indicate a qualitative refinement of the dispersed-phase morphology following reactive compatibilization. Because the present analysis is qualitative, however, the images do not permit the rigorous quantification of dispersed-domain size or interfacial characteristics. Such an analysis would require additional image-based statistical measurements using appropriately prepared specimens. Overall, the SEM observations show clear changes in the phase morphology following the reactive compatibilization and MgO incorporation. However, the present images are used only for qualitative morphological assessment and do not provide a quantitative measure of the MgO dispersion.

### FDM Printability and Processing Performance

This section qualitatively evaluates the FDM printability of the developed formulations rather than the performance of the fully optimized orthopedic devices. Printability was assessed on the basis of filament continuity, dimensional consistency, and visible printing defects, including filament breakage, irregular deposition, and surface voids. Because all formulations were printed under identical processing conditions, the observed differences provided a direct comparison of their relative printability. The results are interpreted in the context of the rheological behavior discussed in the previous section.

Filament quality was first evaluated before printing (Figure 10). The uncompatibilized PHB/PBAT blend (Figure 10a) exhibited noticeable diameter fluctuations and localized surface irregularities, indicating unstable melt flow during extrusion. Incorporation of 0.3 wt % Joncryl produced a more uniform filament with improved continuity and diameter consistency (Figure 10b). The addition of 2.5 wt % MgO maintained this overall filament quality (Figure 10c), whereas 5 wt % MgO introduced localized waviness and surface irregularities (Figure 10d), suggesting that higher filler loading reduced filament uniformity. These observations are consistent with the differences in the melt viscoelasticity identified by rheological characterization.

**Figure 10:**
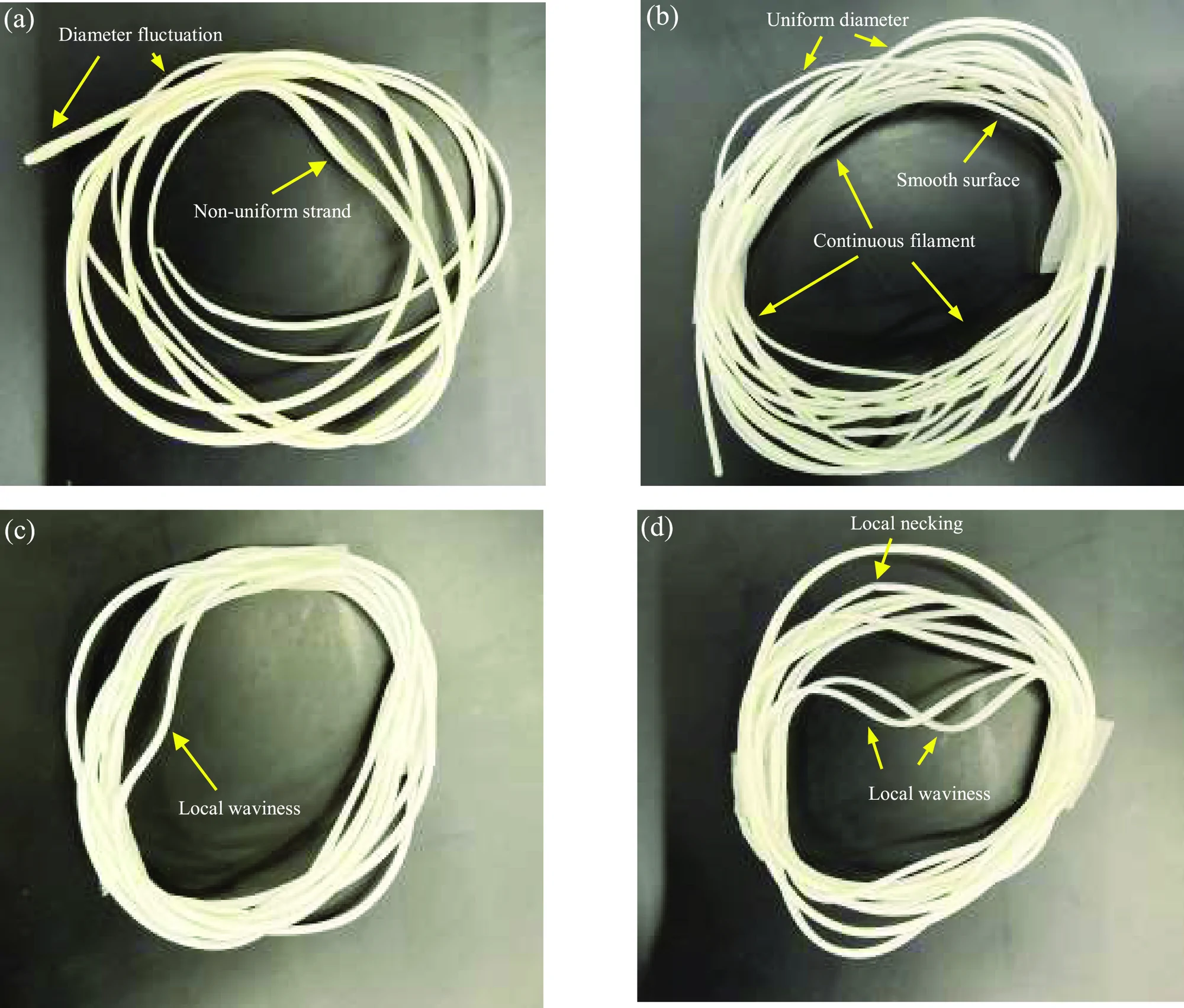
Representative images of extruded filaments of (a) PHB25/PBAT75, (b) PHB25/PBAT75/J0.3, (c) PHB25/PBAT75/J0.3/MgO2.5, and (d) PHB25/PBAT75/J0.3/MgO5, illustrating the effect of composition, reactive compatibilization, and MgO incorporation on filament formation behavior, extrusion stability, dimensional uniformity, and filament integrity.

Representative printed specimens are shown in Figure 11. The incompatible blends exhibited poor layer adhesion, surface roughness, and reduced dimensional stability. Reactive compatibilization progressively improved filament deposition and geometric consistency, whereas excessive MgO loading introduced localized flow instability and reduced the printing quality. These observations indicate that balanced melt viscosity and elasticity are important for maintaining a stable filament deposition under the selected printing conditions.

**Figure 11:**
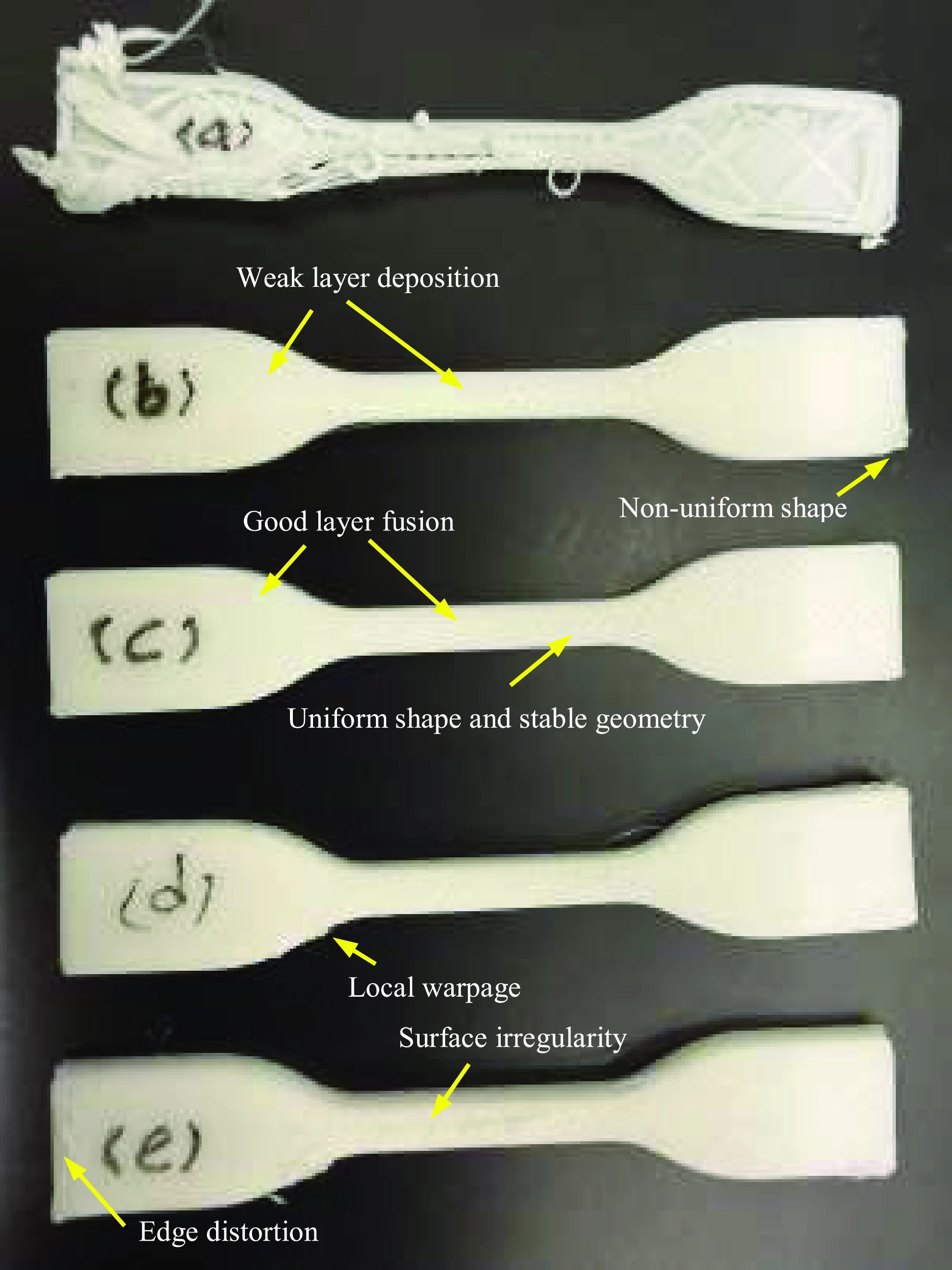
Representative images of FDM-printed dog-bone specimens fabricated from (a) PHB25/PBAT75, (b) PHB25/PBAT75/J0.1, (c) PHB25/PBAT75/J0.3, (d) PHB25/PBAT75/J0.3/MgO2.5, and (e) PHB25/PBAT75/J0.3/MgO5, illustrating the effects of reactive compatibilization and MgO incorporation on printing quality, dimensional uniformity, layer integrity, and defect formation.

To demonstrate the practical printability of the optimized formulation, a representative orthopedic fixation plate was fabricated using the PHB/PBAT/J0.3 filament (Figure 12). The printed structure exhibited continuous filament deposition, acceptable dimensional integrity, and no severe warping or filament collapse. Although only a qualitative assessment was performed, the successful fabrication of this geometry demonstrates that the optimized formulation can produce complex structures under selected printing conditions. Future work will include quantitative evaluation of the dimensional accuracy, surface roughness, interlayer bonding strength, and print-to-CAD deviation.

**Figure 12:**
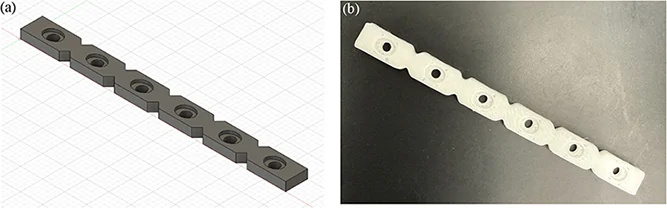
(a) CAD model designed using Fusion 360 and (b) corresponding FDM-printed biodegradable orthopedic fixation plate fabricated from the PHB/PBAT/J0.3 compatibilized blend.

Although biological performance and clinical validation were beyond the scope of this study, the results demonstrated the feasibility of processing the developed biodegradable formulations into complex FDM-printed structures intended for future orthopedic applications.

### Degradation Behavior

The in vitro degradation behavior of PHB/PBAT blends and PHB/PBAT/MgO nanocomposites was evaluated by monitoring mass loss and pH variation during immersion in phosphate-buffered saline (PBS, pH 7.2) at 37 °C. These measurements provide insight into the effect of reactive compatibilization and MgO incorporation on hydrolytic degradation.

Figure 13a Mass loss as a function of immersion time. The PHB/PBAT blends exhibited a gradual mass loss throughout the test period, consistent with hydrolytic degradation of the polyester matrix. The relatively slow initial degradation rate reflects the combined effects of PHB crystallinity and the hydrophobic nature of PBAT, which limits water penetration into the bulk material.

**Figure 13:**
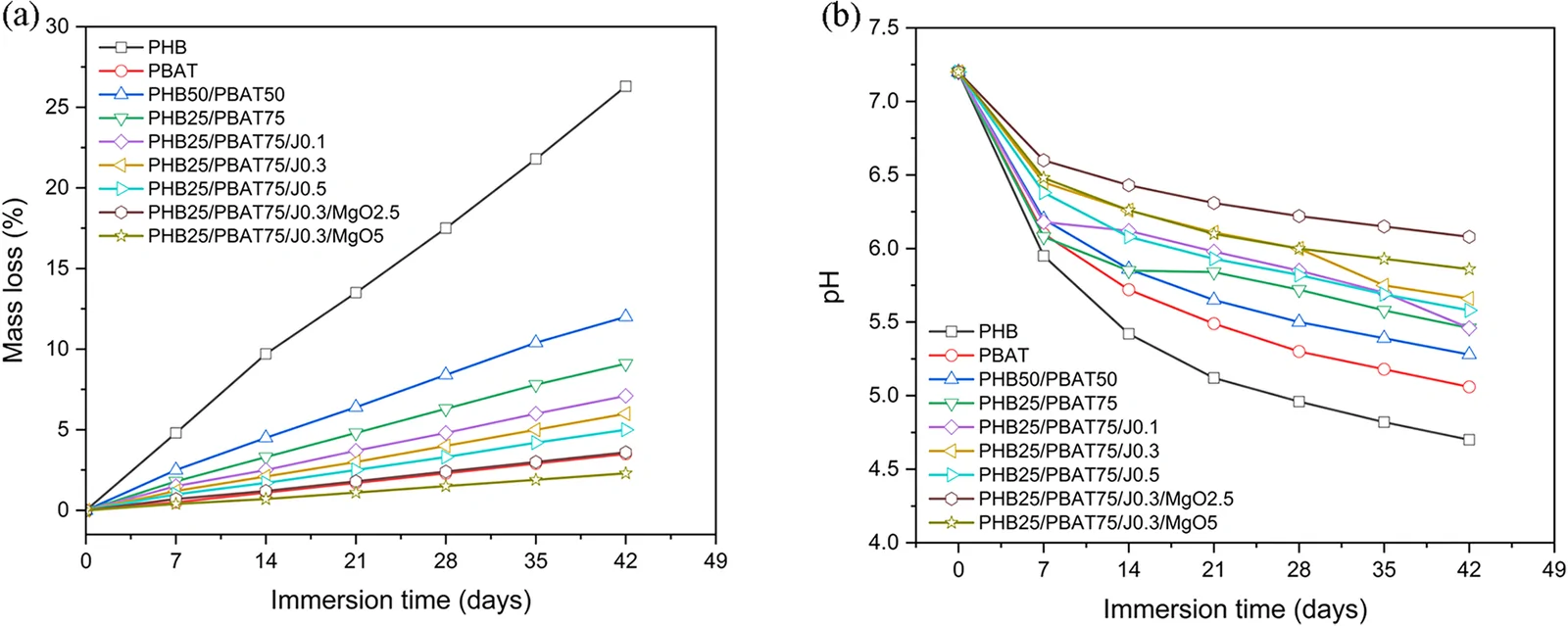
In vitro degradation behavior of neat PHB, PBAT, uncompatibilized and compatibilized PHB/PBAT blends, and PHB/PBAT/Joncryl/MgO nanocomposites in PBS solution at 37 °C: (a) mass loss as a function of immersion time and (b) variation of pH during degradation.

Reactive compatibilization reduced the overall mass loss, indicating a slower degradation rate than that of the uncompatibilized blends. Although the underlying mechanism cannot be established directly from the present results, the observed behavior is consistent with changes in the molecular organization introduced during reactive processing.

The influence of MgO under aqueous conditions differs from its behavior during thermal degradation. At 2.5 wt % MgO, the composites exhibited a more gradual and uniform mass loss than both the MgO-free and higher-MgO formulations. As shown in Figure 13b, the degradation medium also remained closer to neutral pH. These observations are consistent with a buffering effect, whereby MgO may partially neutralize the acidic degradation products generated during polyester hydrolysis. However, this interpretation remains tentative, because Mg^2+^ release, buffering capacity, and local pH within the polymer matrix were not measured directly.

At 5 wt % MgO, the degradation behavior became more heterogeneous. Higher MgO loading may alter local water transport and polymer–filler interactions, leading to spatial variations in hydrolysis and a less uniform mass loss. However, the present results do not allow the specific origin of this heterogeneous degradation behavior to be identified.

In contrast, MgO-free formulations exhibited a greater decrease in the pH of the degradation medium, consistent with the accumulation of acidic degradation products during polyester hydrolysis and the associated autocatalytic degradation processes.

The different effects of MgO under thermal and aqueous conditions can be explained by the dominant degradation mechanisms in each environment. Under nitrogen during TGA, MgO promoted earlier thermal decomposition, whereas under aqueous conditions, it was associated with more controlled degradation and smaller changes in the pH. These results indicate that the role of MgO is environment-dependent rather than contradictory.

The degradation trends are consistent with the surface morphologies shown in Figure 14a–i. Compared with the incompatible blend, the compatibilized formulations exhibited more homogeneous surface erosion and fewer localized defects, indicating improved structural integrity during degradation. Although subtle differences in pore density and surface roughness were observed as the MgO content increased, the SEM analysis remained qualitative. It did not permit quantitative comparison of degradation rates among the investigated formulations.^86–89^

**Figure 14:**
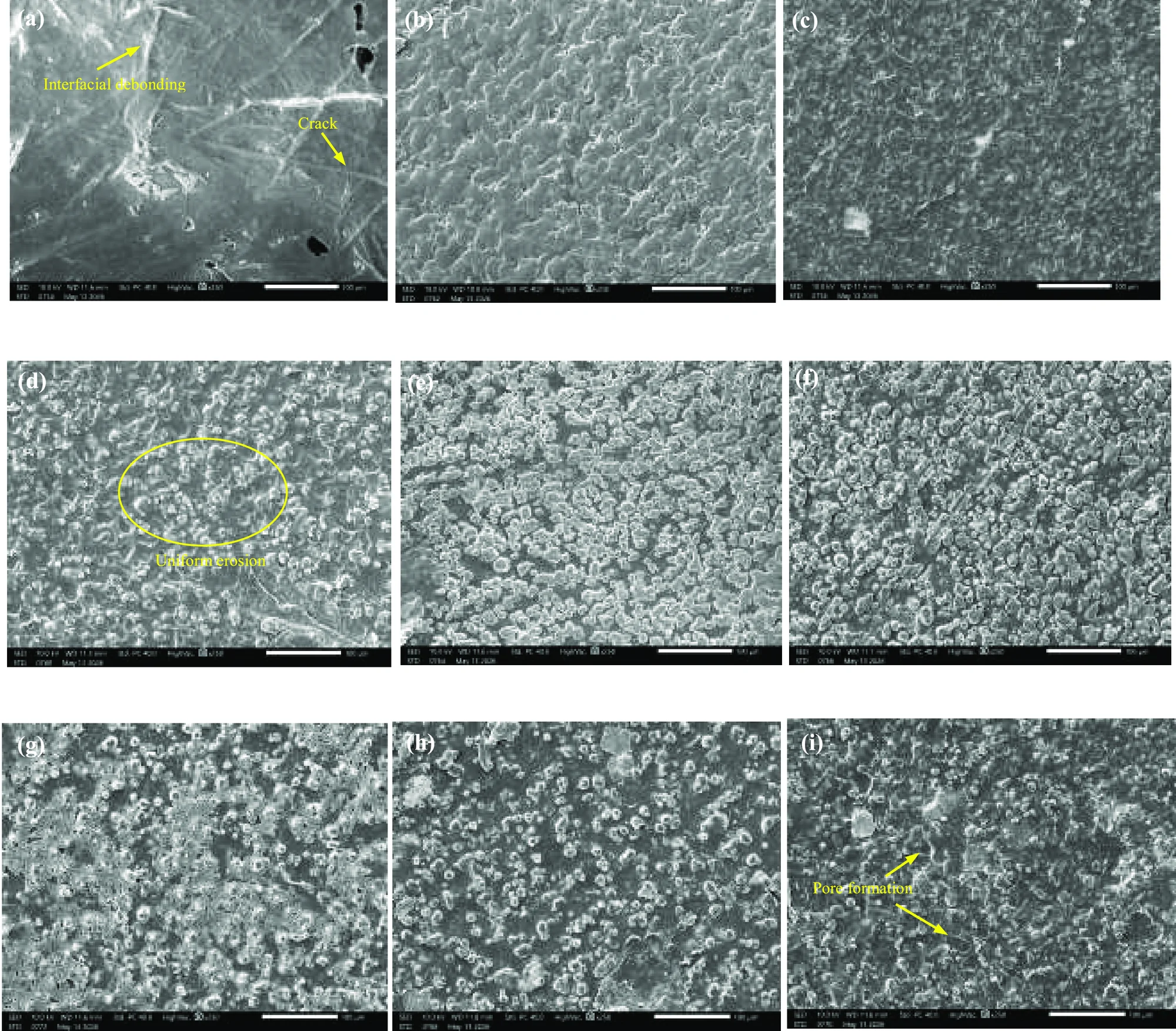
SEM micrographs of degraded surfaces of PHB, PBAT, PHB/PBAT blends, and PHB/PBAT/Joncryl/MgO nanocomposites after 42 days of immersion in PBS solution at 37 °C: (a–d) neat PHB, neat PBAT, PHB75/PBAT25, and PHB25/PBAT75 blends, respectively, and (e–g) compatibilized PHB25/PBAT75 blends with varying Joncryl (0.1, 0.3, and 0.5, respectively), and (h, i) compatibilized PHB25/PBAT75/J0.3 with varying MgO contents (2.5 and 5 wt %, respectively), illustrating changes in surface morphology, erosion behavior, crack formation, and pore development after in vitro degradation.

The incorporation of MgO should therefore be viewed as a functional trade-off rather than as a strategy for maximizing the engineering properties of the PHB/PBAT blend. The present results show that increasing the MgO content reduces several rheological and mechanical properties, including melt viscosity, tensile strength, elongation, and toughness, particularly at higher filler loadings.

These changes indicate that excessive MgO alters the material response in a manner that compromises the mechanical performance achieved through the reactive compatibilization. Nevertheless, MgO provides a distinct functional contribution by modifying the degradation behavior of the material and introducing a magnesium-containing inorganic phase relevant to the intended biodegradable orthopedic applications. Thus, the purpose of MgO incorporation is not to outperform the MgO-free formulation mechanically but to introduce additional functionality while retaining sufficient processability and mechanical integrity for FDM fabrication. From this perspective, the results emphasize the need to optimize MgO loading rather than maximize it, with lower MgO concentrations providing a more favorable compromise between engineering performance and the functional benefits associated with the inorganic phase.

The present study focuses on the effects of reactive compatibilization and MgO incorporation on the degradation behavior in relation to processing and material performance. The biological evaluation of the developed formulations remains the subject of future investigation.

## Conclusions

Biodegradable PHB/PBAT/MgO systems were developed through reactive compatibilization with Joncryl ADR and controlled MgO incorporation for fused deposition modeling (FDM). The results demonstrate that melt rheology provides a useful framework for understanding how formulation influences filament formation, printability, mechanical performance, and degradation behavior in PHB/PBAT-based systems.

Reactive compatibilization increased melt elasticity, storage modulus, complex viscosity, and relaxation time, producing a more stable viscoelastic response during melt processing. These changes were accompanied by improved filament continuity and FDM printability. In contrast, MgO incorporation decreased *G*′ and η*, particularly at higher filler loadings, indicating a more complex modification of the melt response.

Among the investigated formulations, PHB/PBAT/J0.3 provided the most favorable balance of tensile strength, ductility, toughness, and printability. Higher MgO loadings increased the stiffness but reduced the ductility and impact resistance, indicating that excessive filler incorporation compromises the mechanical balance of the system.

Reactive compatibilization also modified crystallization behavior and improved phase morphology, while MgO influenced both thermal and hydrolytic degradation through mechanisms that depended on the degradation environment. The developed formulations exhibited gradual degradation under PBS conditions at 37 °C, supporting their potential for biodegradable applications.

This study demonstrates that optimizing the melt rheology provides an effective strategy for simultaneously improving processability and material performance in biodegradable PHB/PBAT systems. The results highlight the importance of viscoelastic design in developing FDM-printable biodegradable polymers and provide practical guidance for future materials intended for orthopedic and related biomedical applications. Biological performance, including cytocompatibility and long-term in vivo evaluation, remains to be investigated.

## Supplementary Material



## Notes

Enlarged views of selected FTIR spectral regions for PHB/PBAT blends and Joncryl-compatibilized formulations (Figure S1); derivative thermogravimetric analysis (DTGA) curves of PHB, PBAT, PHB/PBAT blends, and PHB/PBAT/Joncryl/MgO nanocomposites (Figure S2); and thermal degradation and DSC parameters, including the calculated crystallinity of the PBAT and PHB phases (Table S1) (PDF)
