# Supplementary material for: Rheology-Guided PHB/PBAT/MgO Nanocomposites for FDM Printing with Balanced Mechanical and Degradation Performance
Source: ACS Omega. 2026 Sep 11;11(37):56566–86. doi: 10.1021/acsomega.6c08431 (PMC13613907; doi:10.1021/acsomega.6c08431)
Supplement: Supplementary file 1 [file ao6c08431_si_001.pdf]

Rheology-Guided PHB/PBAT/MgO Nanocomposites for FDM Printing with Balanced Mechanical and Degradation Performance

Evy Aracely Ortiz[1], Montana Thomas Hance[1], Aboulfazl Barati[1*]

1. *Center for Materials and Manufacturing Sciences, Department of Chemistry and Physics, Troy University, Troy, Alabama, 36082, United States*

**. Corresponding author: abarati@troy.edu*

Supporting Information

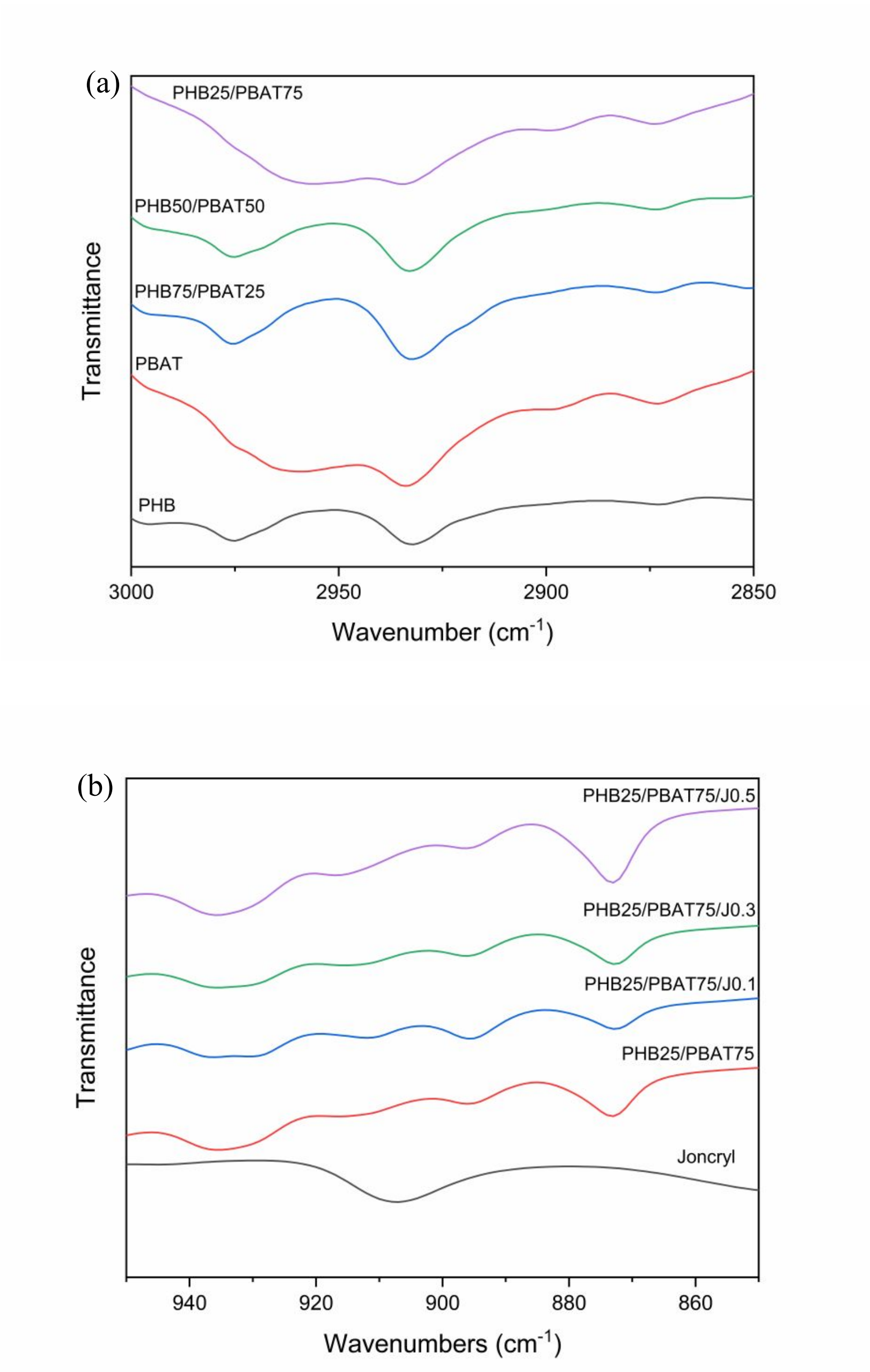

Fig. S1. FTIR spectra of (a) neat PHB, PBAT, and PHB/PBAT blends with different blend ratios, (b) Joncryl and PHB/PBAT (25:75) blends containing different Joncryl contents. Enlarged spectral regions of (a) 3000-2850 cm^{-1}, and (b) 950-800 cm^{-1} are presented to highlight subtle spectral differences among the investigated formulations.

(a)

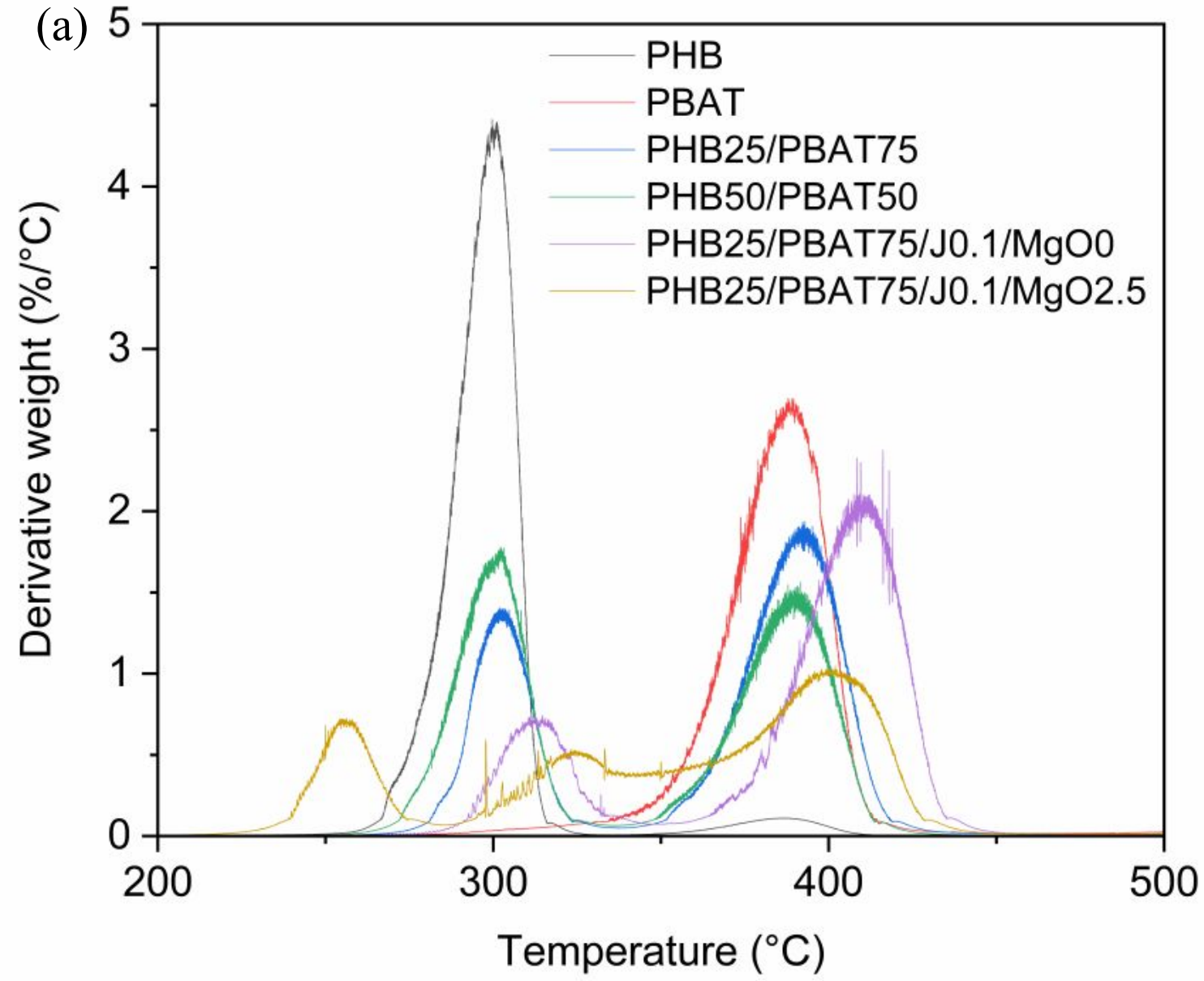

(b)

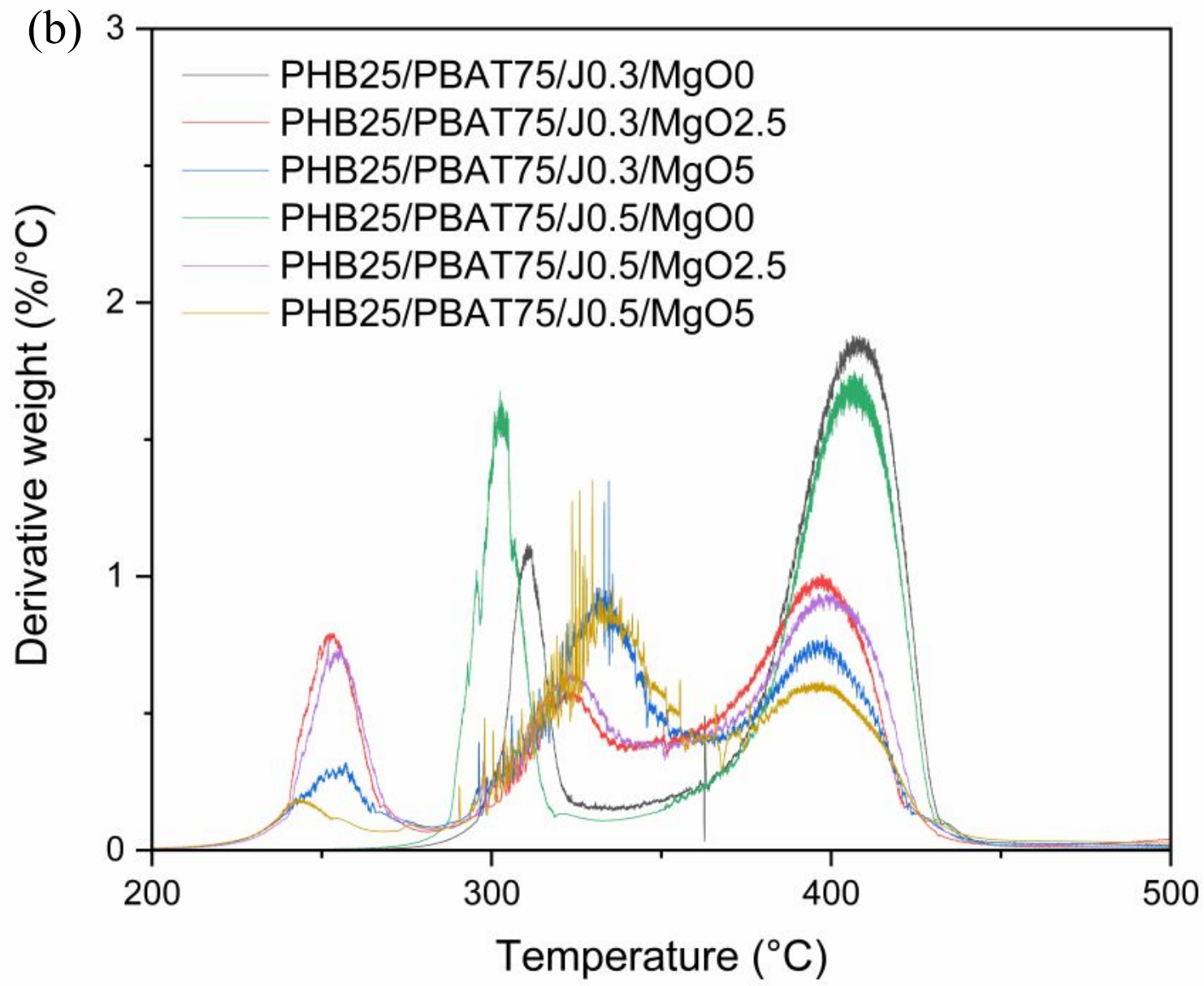

Fig. S2. Derivative thermogravimetric analysis (DTGA) curves of (a) neat PHB, PBAT, and PHB/PBAT blends with different blend ratios, and (b) compatibilized PHB/PBAT/Joncryl/MgO nanocomposites with varying Joncryl and MgO contents under a nitrogen atmosphere. The DTGA profiles highlight the influence of blend composition, reactive compatibilization, and MgO incorporation on degradation pathways and decomposition peak temperatures of the biodegradable polymer systems.

Table S1: Thermal degradation and DSC parameters of PHB, PBAT, uncompatibilized and compatibilized PHB/PBAT blends, and PHB/PBAT/Joncryl/MgO nanocomposites. *

Sample	ΔH_c (PBAT)	T_c (°C) (PBAT)	ΔH_c (PHB)	T_c (°C) (PHB)	ΔH_m (PBAT)	T_m (°C) (PBAT)	ΔH_m (PHB)	T_m (°C) (PHB)	T_g (°C)	$T_{5\%}$ (°C)	X_c (PBAT)	X_c (PHB)
PHB	---	---	31.05 ± 0.7	38.37 ± 0.1	---	---	63.28 ± 0.7	165.57 ± 0.1	-7.25 ± 0.1	276 ± 0.3	---	0.4343 ± 0.010
PHB/PBAT 75:25	3.98 ± 0.5	66.60 ± 0.1	25.25 ± 0.6	42.57 ± 0.1	2.48 ± 0.5	119.12 ± 0.1	39.39 ± 0.6	165.76 ± 0.1	-6.72 ± 0.1	279 ± 0.3	0.0870 ± 0.01	0.3634 ± 0.010
PHB/PBAT 50:50	7.41 ± 0.5	66.88 ± 0.1	11.89 ± 0.5	41.80 ± 0.1	4.95 ± 0.5	119.85 ± 0.1	25.93 ± 0.5	166.00 ± 0.1	-6.60 ± 0.1	283 ± 0.3	0.0868 ± 0.01	0.3588 ± 0.010
PHB/PBAT 25:75	12.26 ± 0.6	65.83 ± 0.1	3.93 ± 0.5	43.73 ± 0.1	8.25 ± 0.6	119.80 ± 0.1	6.36 ± 0.5	166.69 ± 0.1	-5.76 ± 0.1	292 ± 0.3	0.0964 ± 0.01	0.1760 ± 0.020
PBAT	17.62 ± 0.6	69.02 ± 0.1	---	---	11.51 ± 0.6	120.07± 0.1	---	---	---	348 ± 0.5	0.1009 ± 0.01	---
PHB/PBAT/J 25:75:0.1	12.73 ± 0.6	74.60 ± 0.1	0.59 ± 0.1	45.51 ± 0.1	7.78 ± 0.5	122.9 ± 0.1	0.98 ± 0.1	167.45 ± 0.1	-5.25 ± 0.1	304 ± 0.4	0.0909 ± 0.01	0.0272 ± 0.001
PHB/PBAT/J/MgO 25:75:0.1:2.5	12.29 ± 0.6	74.39 ± 0.1	---	---	8.36 ± 0.6	122.28 ± 0.1	0.13 ± 0.1	156.57 ± 0.1	-5.57 ± 0.1	250 ± 0.3	0.0977 ± 0.01	0.0037 ± 0.001
PHB/PBAT/J/MgO 25:75:0.1:5	11.67 ± 0.6	70.65 ± 0.1	---	---	8.57 ± 0.5	121.52 ± 0.1	0.42 ± 0.1	157.76 ± 0.1	-5.24 ± 0.1	255 ± 0.3	0.1002 ± 0.02	0.0123 ± 0.003
PHB/PBAT/J 25:75:0.3	13.09 ± 0.6	72.49 ± 0.1	---	---	9.23 ± 0.6	123.30 ± 0.1	0.31 ± 0.1	168.11 ± 0.1	-3.75 ± 0.1	307 ± 0.4	0.1079 ± 0.01	0.0086 ± 0.003
PHB/PBAT/J/MgO 25:75:0.3:2.5	12.12 ± 0.5	71.45 ± 0.1	---	---	8.69 ± 0.5	122.49 ± 0.1	---	---	-4.66 ± 0.1	247 ± 0.3	0.1016 ± 0.02	---
PHB/PBAT/J/MgO 25:75:0.3:5	13.47 ± 0.5	68.65 ± 0.1	---	---	9.26 ± 0.6	121.23 ± 0.1	---	---	-4.92 ± 0.1	254 ± 0.3	0.1083 ± 0.01	---
PHB/PBAT/J 25:75:0.5	12.47 ± 0.5	73.87 ± 0.1	1.96 ± 0.1	50.41 ± 0.1	7.84 ± 0.5	122.92 ± 0.1	3.45 ± 0.1	166.86 ± 0.1	-4.49 ± 0.1	295 ± 0.3	0.0916 ± 0.01	0.0960 ± 0.003
PHB/PBAT/J/MgO 25:75:0.5:2.5	13.11 ± 0.6	72.17 ± 0.1	---	---	8.85 ± 0.6	121.92 ± 0.1	---	---	-4.84 ± 0.1	249 ± 0.3	0.1035 ± 0.01	---
PHB/PBAT/J/MgO 25:75:0.5:5	12.64 ± 0.5	68.62 ± 0.1	---	---	9.15 ± 0.6	121.07 ± 0.1	---	---	-5.29 ± 0.1	262 ± 0.3	0.1070 ± 0.01	---

*. ΔH_c and ΔH_m have the unit of J g^{-1}.
